# Plasticity of the Chemoreceptor Repertoire in *Drosophila melanogaster*

**DOI:** 10.1371/journal.pgen.1000681

**Published:** 2009-10-09

**Authors:** Shanshan Zhou, Eric A. Stone, Trudy F. C. Mackay, Robert R. H. Anholt

**Affiliations:** 1 Department of Biology, North Carolina State University, Raleigh, North Carolina, United States of America; 2 W. M. Keck Center for Behavioral Biology, North Carolina State University, Raleigh, North Carolina, United States of America; 3 Department of Statistics, North Carolina State University, Raleigh, North Carolina, United States of America; 4 Department of Genetics, North Carolina State University, Raleigh, North Carolina, United States of America; The Wellcome Trust Centre for Human Genetics, University of Oxford, United Kingdom

## Abstract

For most organisms, chemosensation is critical for survival and is mediated by large families of chemoreceptor proteins, whose expression must be tuned appropriately to changes in the chemical environment. We asked whether expression of chemoreceptor genes that are clustered in the genome would be regulated independently; whether expression of certain chemoreceptor genes would be especially sensitive to environmental changes; whether groups of chemoreceptor genes undergo coordinated rexpression; and how plastic the expression of chemoreceptor genes is with regard to sex, development, reproductive state, and social context. To answer these questions we used *Drosophila melanogaster*, because its chemosensory systems are well characterized and both the genotype and environment can be controlled precisely. Using customized cDNA microarrays, we showed that chemoreceptor genes that are clustered in the genome undergo independent transcriptional regulation at different developmental stages and between sexes. Expression of distinct subgroups of chemoreceptor genes is sensitive to reproductive state and social interactions. Furthermore, exposure of flies only to odor of the opposite sex results in altered transcript abundance of chemoreceptor genes. These genes are distinct from those that show transcriptional plasticity when flies are allowed physical contact with same or opposite sex members. We analyzed covariance in transcript abundance of chemosensory genes across all environmental conditions and found that they segregated into 20 relatively small, biologically relevant modules of highly correlated transcripts. This finely pixilated modular organization of the chemosensory subgenome enables fine tuning of the expression of the chemoreceptor repertoire in response to ecologically relevant environmental and physiological conditions.

## Author Summary

Rapid adaptation and phenotypic plasticity to the chemical environment are essential prerequisites for survival; and, consequently, large families of genes that mediate the recognition of olfactory and gustatory cues have evolved. We asked how flexible the expression of these genes is in the face of rapidly changing conditions encountered during an individual's lifetime. We used the fruit fly, *Drosophila melanogaster*, to address this question, since both the genetic composition and environmental rearing conditions can be controlled precisely in this experimentally amenable model organism. By measuring expression levels of all chemosensory genes simultaneously, we identified genes that show altered expression at different developmental stages, during aging, in males and females, following mating, and in different social conditions. We asked whether chemosensory genes are regulated independently or whether their regulation is structured. We found that chemosensory genes that are located in close proximity to one another on the chromosome are often regulated independently. However, statistical analysis showed that groups of chemosensory genes are coordinately expressed in response to a range of environmental conditions, revealing an underlying modular organization of the phenotypic plasticity of the chemosensory receptor repertoire.

## Introduction

Responses to the chemical environment play an important role in animal survival, as chemical cues direct foraging behavior and food selection, predator avoidance, and, in insects, host plant recognition for oviposition and larval feeding. Chemical signals are also essential for the selection of mating partners, maternal behavior, and kin recognition. As a consequence of the profound importance of chemosensation for survival and reproduction, several large families of chemosensory genes have evolved through repeated processes of gene duplication and diversification [1]–[4], including genes that encode odorant receptors (*Or*s) [4]–[8], gustatory receptors (*Gr*s) [4],[9], and, in insects, odorant binding proteins (*Obp*s) [10]–[12]. In addition, large multigene families aimed at eliminating toxic chemicals have evolved, most prominently the cytochrome P450 superfamily [13]. Detoxification of plant defense chemicals together with development of chemosensors that enable fine tuning to host plants has been instrumental in the establishment of specialized insect-host plant relationships [14]. For example, the black swallowtail butterfly, *Papilio polyxenes*, has developed cytochrome P450s that can metabolize toxic furanocoumarins, which allows it to feed and oviposit on plants of the Umbelliferae family [15]. Similarly, *Drosophila sechellia*'s host plant, *Morinda citrifolia*, is toxic to other Drosophila species. A 4 bp insertion in the upstream regulatory region of the *D. sechellia Obp57e* gene eliminates expression of this odorant binding protein, which elicits avoidance of the Morinda fruit in Drosophila species in which the gene is intact [16].

The rapid evolution of these large chemoreceptor gene families has generated functional redundancy between receptors and their ligands [17],[18], which confers sensitivity and robustness to the chemical recognition process. Animals, however, interact differently with their chemosensory environments under different developmental, physiological and social conditions. Therefore, it stands to reason that expression of the chemosensory repertoire would be dynamically regulated. This raises several fundamental questions: (1) Is the expression of chemoreceptor genes that are organized as clusters in the genome independently regulated or do genes within a cluster act as co-regulated functional ensembles? (2) Are all chemoreceptor genes equally sensitive to environmental fluctuations or is a core group of chemoreceptor genes particularly responsive to environmental or physiological changes? (3) Are certain chemoreceptor genes frequently co-regulated when environmental or physiological conditions change? (4) Is the expression of particular chemoreceptor genes upregulated or downregulated as a function of sex (males *versus* females), development (*e.g.* in larval stages, adult stages and aged flies), reproductive state (*e.g.* virgin or mated) or social context (*e.g.* solitary or group reared)?

To answer these questions we focused on the chemoreceptor families of *Drosophila melanogaster*, where both the olfactory and gustatory systems have been well characterized [4], [6]–[12],[19]. *D. melanogaster* provides an advantageous genetic model as inbred individuals can be readily generated and grown under controlled conditions, enabling control over both the genotype and the environment [20]. We constructed expression microarrays that enable us to survey simultaneously expression of all *Obp*, *Or* and *Gr* genes. We analyzed chemoreceptor expression as a function of sex, development, reproductive state, and social environment, and obtained a systematic description of the plasticity of the chemosensory window through which the fly experiences its chemical environment. We found that genes in clusters are independently regulated in the two sexes, during different developmental stages, and under different physiological and social conditions. Whereas many chemosensory genes showed plasticity in expression, a smaller number of exceptionally plastic genes was evident. Analysis of covariance of transcript levels across all environmental conditions showed that the chemosensory subgenome is structured as a mosaic of 20 small modules of highly correlated transcripts. This finely pixilated modular organization of the chemosensory transcriptome allows finely tuned phenotypic plasticity of expression of the chemoreceptor repertoire under different environmental conditions.

## Results

### Construction and Characterization of the cDNA Microarrays

To assess to what extent transcription of chemosensory genes responds to changing conditions, we constructed cDNA expression arrays that represent 50 *Odorant binding protein* (*Obp*), 59 *Odorant receptor* (*Or*), and 59 *Gustatory receptor* (*Gr*) genes, four genes that encode other antenna-specific proteins, and four control genes. To prepare cDNA probes, primer sets were designed to generate unique 400–600 bp amplicons. All amplification products were sequenced and the sequences analyzed using the BLAST algorithm to ensure absence of cross-hybridizing sequences. Cross-hybridization is likely to occur in only two cases. Amplicons for *Gr64d* and *Gr64e* do not overlap, but these genes have partially overlapping transcripts and, therefore, could cross-hybridize. In addition, *Or19a* and *Or19d* are located 50 kb apart in opposite orientation and share the same sequences, rendering them indistinguishable. The extent of dye effects was assessed by hybridization of a mixture of equal amounts of Cy3 and Cy5 labeled RNA of the same sample. There was generally a close correlation between Cy3 and Cy5 hybridization intensities (Figure S1), indicating overall minor dye effects.

Among the 168 chemosensory genes represented on the microarray, we detected expression of 50 *Obp* genes, 54 *Or* genes, and 52 *Gr* genes, in at least one experimental condition. Expression levels of *Obp* genes were generally at least one order of magnitude higher than those of *Or* and *Gr* genes. Expression of chemoreceptor genes on our customized EST microarrays correlated well with previously obtained transcriptional profiles of chemosensory genes represented on high density oligonucleotide microarrays from Affymetrix, Inc. [21] ((Figure S2; *r* = 0.818, *n* = 174), but resolution for detection of chemoreceptor gene expression was substantially improved. We were not able to detect expression of *Gr22b*, *Gr58c*, *Gr59c*, *Gr77a*, *Gr93b*, *Gr93c*, *Gr93d*, *Or10a*, *Or24a*, *Or85b*, *Or85c* and *Or85d*, possibly due to highly localized expression of rare transcripts.

### Modulation of Chemoreceptor Gene Expression during Development

**Figure 1:**
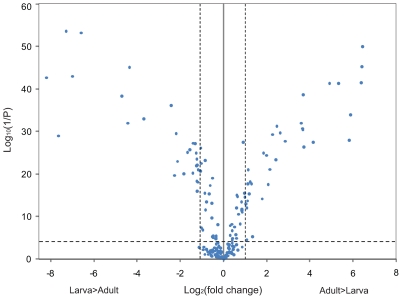
Volcano plot of differences in transcript abundance between larvae and adult flies. The figure illustrates differences in transcript expression levels between RNA extracted from third instar larvae and from an equal mixture of virgin adult males and females. Each dot represents a probe on the array. The horizontal dashed line shows the Bonferroni-corrected significance threshold of *P* = 5.2E-5. The vertical dashed lines show 2-fold enrichment boundaries between the samples.

**Figure 2:**
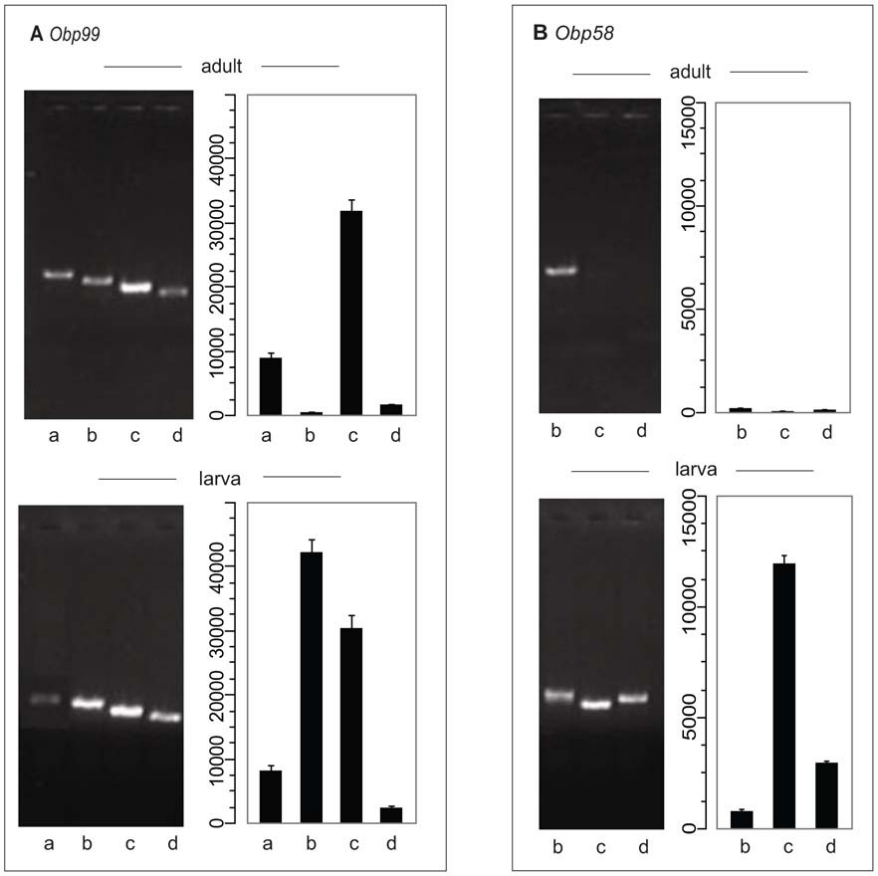
Confirmation of microarray expression data by RT–PCR. Fragments of cDNA corresponding to transcripts of *Obp* genes within the *Obp99* (A) and *Obp58* (B) clusters were amplified from adult (top panels) or larval (bottom panels) RNA samples using the same primers used to construct the corresponding microarray probes. Amplification was done after 2 min denaturation at 94°C by 30 s denaturation at 94°C, 30 s annealing at 55°C, and 1 min extension at 72°C for 30 cycles followed by 4 min incubation at 72°C. Intensity of ethidium bromide stained bands on agarose gels are compared to fluorescence intensities after hybridization of labeled RNA samples to the microarrays (bar graphs in each panel). Quantitative comparisons are not precise at low levels of expression. Note, however, that absence of *Obp58c* in adults and high intensities of *Obp58c* in larvae on the arrays are matched by the appearance of the corresponding RT–PCR products. Similarly, high intensity levels of *Obp99c* in adults and *Obp99b* and *Obp99c* in larvae correspond between fluorescent intensity levels on the microarrays and staining of the corresponding RT–PCR products.

To assess modulation of chemoreceptor gene expression during development we compared expression of *Obp*, *Or* and *Gr* genes in third instar larvae (mixed sexes) and in virgin adult males and females. We also assessed changes in chemoreceptor gene expression in aged males and females. Pairwise comparisons between larvae and adults showed that relative expression of 28 chemoreceptor genes was biased in or specific to larvae at a Bonferroni corrected significance threshold of *P*<5.68E-5 (corrected for multiple testing at a nominal significance level of *P*<0.01) with a 2-fold change filter; conversely, 35 chemoreceptor genes showed adult-biased or adult-specific relative expression (Figure 1; Table S1). To validate our microarray observations, we amplified transcripts of the *Obp58* and *Obp99* gene clusters in larvae and adults. *Obp99c* was highly expressed in larvae and adults, whereas *Obp99b* showed strong adult-biased expression (Figure 2). Similarly, *Obp58c* and *Obp58d* were virtually undetectable in larvae, but expressed in adults with especially strong adult-specific expression of *Obp58c*. The results of the microarray analysis showed good concordance with results from RT-PCR experiments (Figure 2).

**Figure 3:**
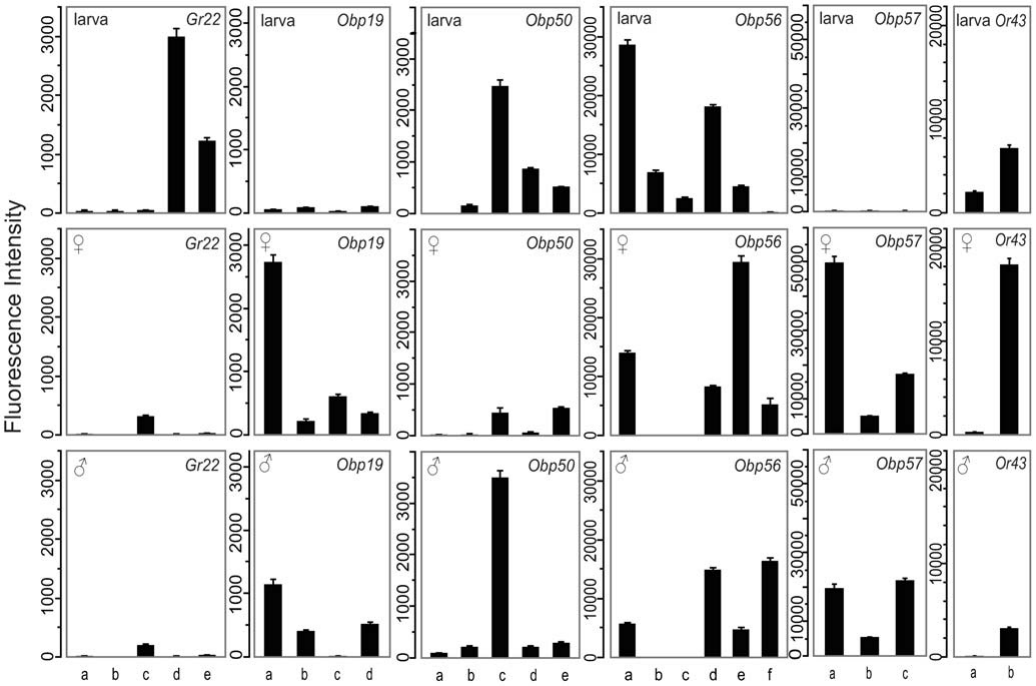
Differential and sexually dimorphic expression of chemoreceptor genes between larvae and adults. Average fluorescent intensities are shown corresponding to expression of chemosensory genes within the *Gr22*, *Obp19*, *Obp50*, *Obp56*, *Obp57*, and *Or43* gene clusters in larvae (top panels), adult virgin females (center panel), and adult virgin males (bottom panels). None of the clusters shown contain intervening genes. Note the dramatic differences in expression patterns between larvae and adults with strict larva-specific expression of *Gr22d*, *Obp56b*, and *Obp56c*, the extensive sexual dimorphism among adults, and the apparently independent regulation among genes within the same cluster.

Since many chemoreceptors occur in clusters in the genome [4], we asked whether individual members of a cluster show coordinated or independent rexpression during development. We examined chemoreceptor gene clusters without intervening genes, including the *Gr22a–e* cluster, the *Obp19a–d*, *Obp50a–e*, *Obp56a–f*, and *Obp57a–c* clusters, and the *Or43a–b* cluster (Figure 3). There were extensive differences between larvae and adults in chemoreceptor gene expression. *Gr22d*, *Gr22e*, *Obp50d*, *Obp56a–d*, and *Or43a* showed larva-biased expression. Especially striking was the high larva-specific expression of *Gr22d*, as well as *Gr22e*. In contrast, expression of some chemoreceptor genes was observed only in adults, for example the *Obp19a–d* and *Obp57a–c* gene clusters and *Obp56f*.

When we compared relative expression of the same chemoreceptor genes in males and females, we observed extensive sexual dimorphism in transcript abundance levels. Male-biased expression was evident for *Obp50c*, *Obp56d*, and *Obp56f*, whereas female-biased expression was observed for *Obp19a*, *Obp19c*, *Obp56a*, *Obp56e*, *Obp57a*, and *Or43b* (Figure 3; Table S2). These results show that expression of chemoreceptor genes that are located within gene clusters can be regulated independently at different developmental stages and between the sexes.

**Figure 4:**
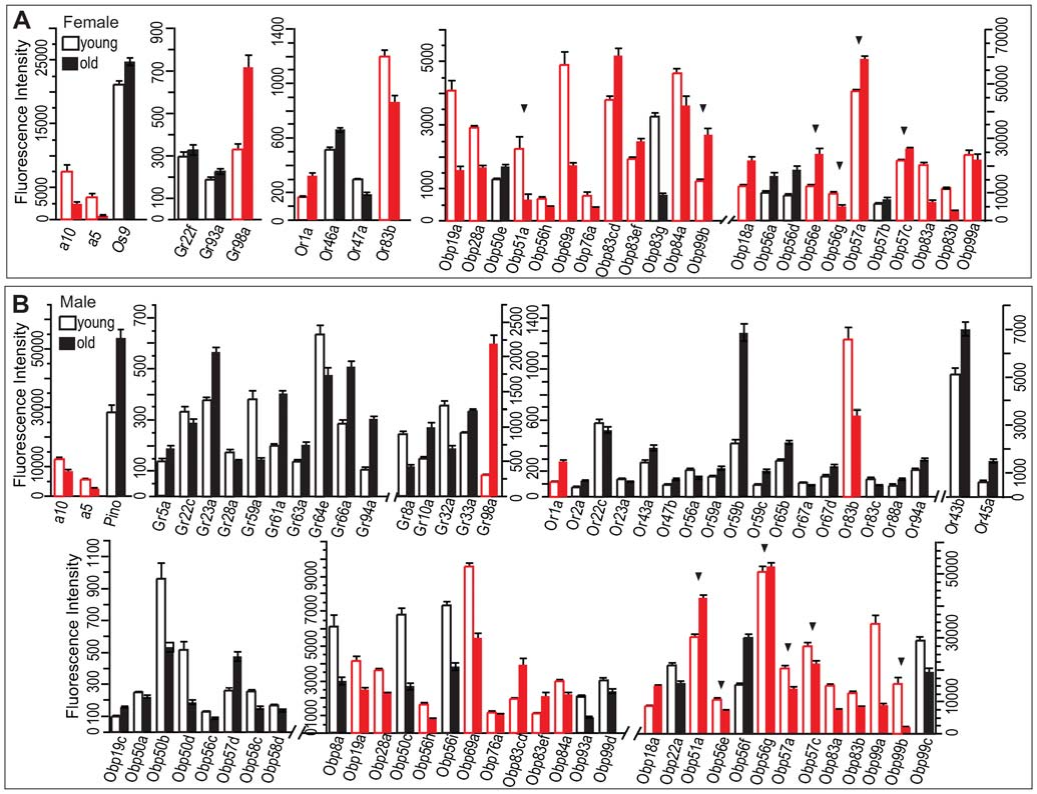
Differential expression of chemoreceptor genes during ageing. Only chemoreceptor genes of which expression levels change significantly after Bonferroni correction between 7–10 day-old (open bars) and 6 week-old (solid bars) virgin females (A) or males (B) are shown. Red bars indicate chemoreceptor genes with altered expression in aged flies in both sexes. Note the preponderance of *Obp* genes. Arrowheads indicate chemoreceptor genes of which expression changes in opposite directions between males and females (*Obp51a*, *Obp99b*, *Obp56e*, *Obp57a*, and *Obp57c*).

Next, we asked whether chemosensory gene expression levels are stable throughout adult live or are subject to age-dependent plasticity. We compared transcript abundance levels in 10-day old and 6-week-old virgin males and females maintained under carefully controlled standard laboratory conditions, and found extensive age-dependent changes in transcript abundance in all classes of chemosensory genes (Figure 4). We found 104 chemosensory genes with altered transcript abundance in one or both sexes. Many genes with altered expression in aged flies were shared between males and females. However, sexual dimorphism in age-dependent chemoreceptor gene expression was pervasive. Interestingly, in males 15 *Gr* genes and 19 *Or* genes showed alterations in expression levels during aging (Figure 4B), while in females only three *Gr* genes and four *Or* genes changed expression levels during ageing (Figure 4A). The ubiquitous odorant receptor *Or83b* showed decreased expression levels with age in both sexes, whereas expression of *Or1a* and *Gr98a* was upregulated in both sexes during aging. Extensive differences among transcript abundance levels of *Obp* genes in young and old flies were especially prevalent for both sexes. *Obp51a*, *Obp56e*, *Obp56g*, *Obp57a*, *Obp57c*, and *Obp99b* showed altered expression levels during aging in both sexes, but in opposite directions (Figure 4). Again, expression of genes within a cluster appears to be regulated independently from other genes in the same cluster during ageing.

### Modulation of Chemoreceptor Gene Expression by Reproductive State

**Figure 5:**
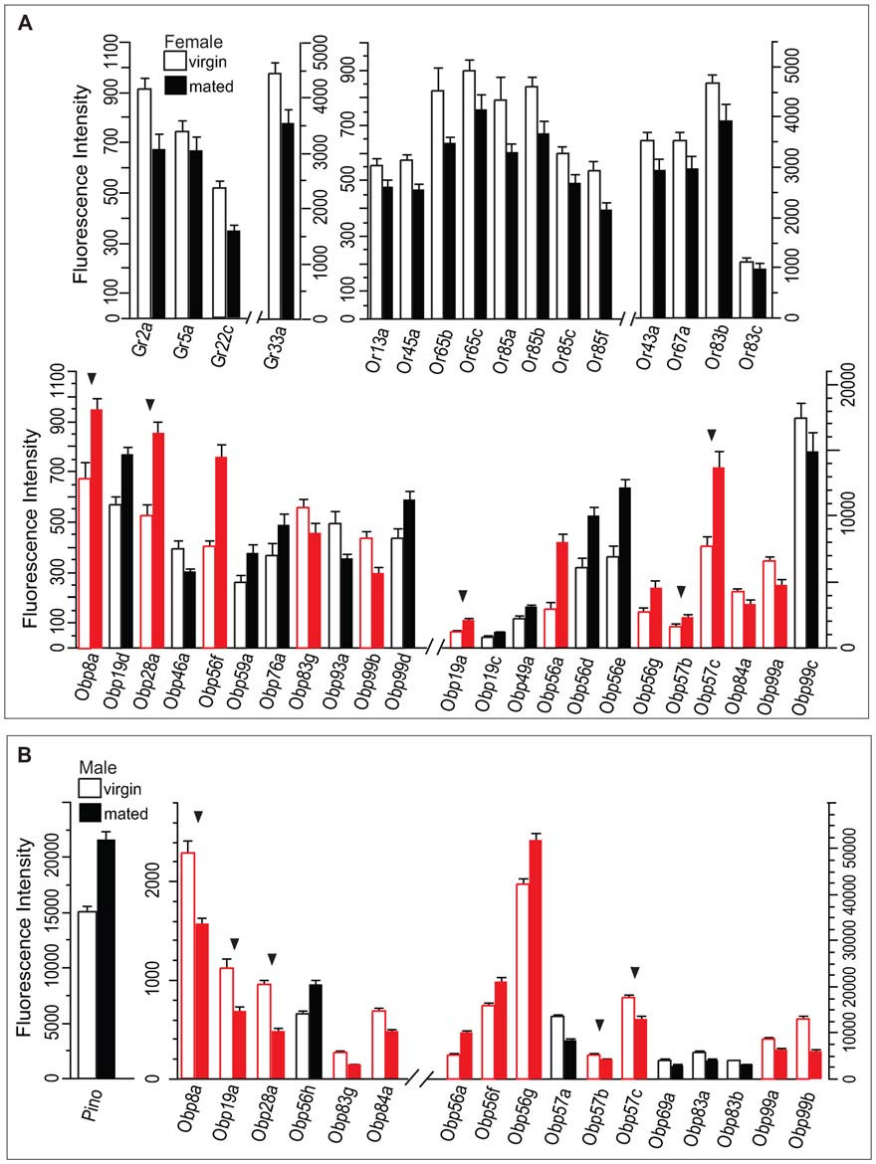
Differential expression of chemoreceptor genes after mating. Only chemoreceptor genes of which expression levels change significantly after Bonferroni correction between virgin (open bars) and mated (solid bars) females (A) or males (B) are shown. Red bars indicate chemoreceptor genes with altered expression after mating in both sexes. Note the preponderance of *Obp* genes. Differential expression of *Gr* and *Or* genes after mating is only observed in females. Arrowheads indicate chemoreceptor genes of which expression changes in opposite directions between males and females (*Obp8a*, *Obp19a*, *Obp57b*, and *Obp57c*).

Next, we asked to what extent changes in physiological condition affect expression of the chemoreceptor repertoire. Mating results in physiological changes in females [22] and males [23]–[25]. We compared transcript abundance levels of chemosensory genes in virgin males and females reared separately to those of individuals that were allowed to mate (Figure 5). Following mating, only females showed a reduction in transcript levels of a suite of four *Gr* and 12 *Or* genes. In contrast, changes in *Obp* transcript abundance were seen in both sexes. Here, 16 out of 23 *Obp* genes with altered transcript abundance showed up-regulation in mated females (Figure 5A). Substantial changes in transcript abundance of *Obp* genes and *Pino* (a.k.a. *smi21F*), a putative odorant binding protein [26], were also evident in mated males (Figure 5B). Twelve *Obp* genes showed altered expression in both sexes, and among these five showed antagonistic changes in expression levels between the sexes (Figure 5). Thirteen out of 19 *Obp* genes with altered transcript abundance in mated males showed a reduction in transcript abundance, in contrast to the predominant up-regulation of *Obp* expression levels seen in mated females. Thus, mating caused profound changes in subsets of chemosensory genes in both sexes. The identities of the chemosensory genes affected or the effect on their transcript levels were distinct between males and females, indicating a profound sexually dimorphic change in the functional composition of the chemoreceptor repertoire after mating.

### Modulation of Chemoreceptor Gene Expression by Social Context

**Figure 6:**
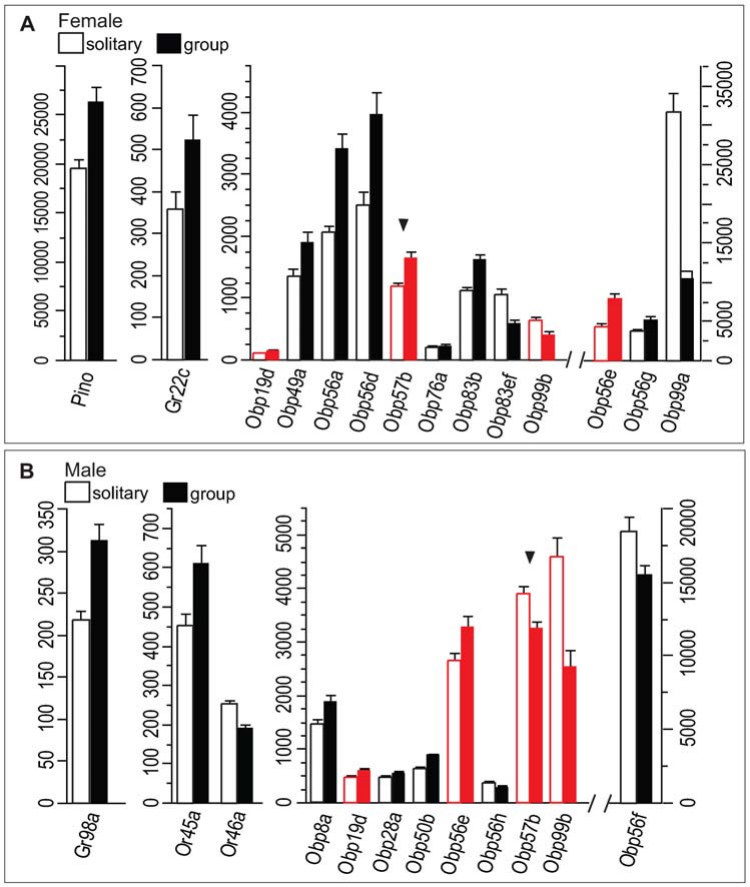
Regulation of chemoreceptor gene expression by social context. Only chemoreceptor genes of which expression levels change significantly after Bonferroni correction when flies are reared solitary (open bars) or in same sex groups (solid bars) are shown for females (A) and males (B). Note the preponderance of *Obp* genes. Red bars indicate Obp56e and *Obp57b* which show altered expression in different social contexts in both sexes. Arrowheads indicate chemoreceptor genes of which expression changes in opposite directions between males and females in opposite directions.

Our observation that the expression of the chemosensory repertoire is modified dramatically by social contact during reproduction led us to ask whether social context *per se* can elicit altered expression of distinct chemosensory genes. We compared transcript abundance levels in male and female flies that were reared as single isolated individuals to those of virgin flies reared in corresponding single sex groups. We observed changes in expression levels of few *Gr* or *Or* genes under these conditions (Figure 6). However, in females transcript abundance levels of seven *Obp* genes and *Pino* were down-regulated when individuals were reared in isolation, whereas two *Obp* genes were up-regulated (Figure 6A). In males transcription of five *Obp* genes was down-regulated when individuals were reared in isolation, whereas three *Obp* genes were upregulated (Figure 6B). Compared to our other experimental conditions, we found less overlap between genes with altered transcript abundance in males and females. Different members of the *Obp56* gene cluster featured prominently among transcripts with altered levels in each sex. Only *Obp56e*, however, showed down-regulation in isolated individuals in both sexes and *Obp57b* was down-regulated in females and up-regulated in males when flies were reared in isolation (Figure 6).

**Figure 7:**
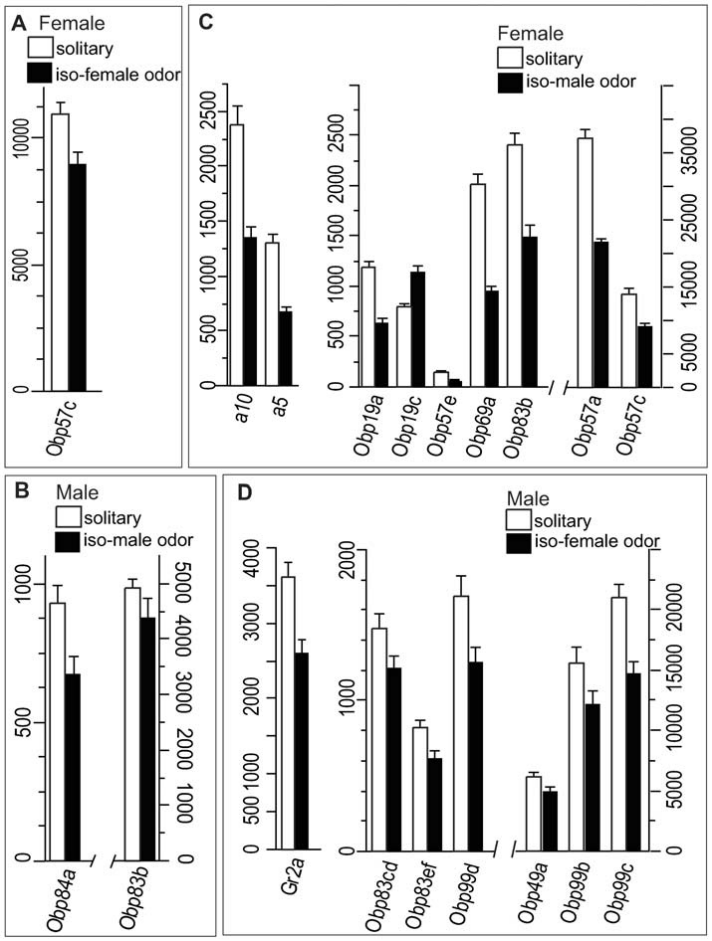
Social odor induced differential expression of chemoreceptor genes. Only chemoreceptor genes of which expression levels change significantly after Bonferroni correction between single fly controls (solid bars) and single flies exposed to same sex odor or opposite sex odor (open bars) are shown for females ((A), exposed to female group odor; and (C), exposed to male group odor) and males ((B), exposed to male group odor; and (D), exposed to female group odor). Red bars indicate *Obp83b* which shows altered expression in both sexes when single flies are exposed to male group odor, but in opposite directions between the sexes (arrowhead). Note that differential chemoreceptor gene expression is more prominent when flies are exposed to opposite sex group odor than to same sex group odor.

Chemoreceptors have been implicated in the detection of both volatile [27] and non-volatile [28] social chemical signals. We wanted to assess whether exposure to social odor cues alone could result in altered transcript abundance of chemosensory genes. Therefore, we separated single flies from groups of same-sex or opposite sex flies with a double cheesecloth partition that would allow the transmission of olfactory cues, but would prevent physical interaction (it should be noted that *Canton S w^−^* flies used in these experiments are visually impaired). When single flies were maintained for five days under conditions in which they were exposed to same-sex group odors, there were virtually no changes in transcript patterns of chemosensory genes. Only expression of *Obp57c* was increased in females exposed to female group odor (Figure 7A), whereas expression of *Obp84a* and *Obp83b* was increased in males exposed to male group odor (Figure 7B). In contrast, we saw more extensive changes in transcript levels when we exposed single flies to opposite sex group odor for the same time period. Here, nine chemosensory genes in females showed altered transcript levels, including seven *Obp* genes and the antenna-specific *a5* and *a10* genes (Figure 7C). With the exception of *Obp19c*, all of these genes were down-regulated when a single female was exposed to male group odor. In single males exposed to female group odor, six *Obp* genes and a gustatory receptor gene (*Gr2a*) showed altered transcript levels (Figure 7D). Remarkably, there was no overlap between the subsets of chemosensory genes that had altered transcript levels when single males or females were exposed to opposite sex group odor. Notably, members of the *Obp56* gene cluster (Figure 6) did not show altered expression under these conditions. The lack of concordance between transcript abundance of chemosensory genes when isolated individuals were compared to group reared individuals (Figure 6) and when isolated individuals were limited only to same sex group olfactory exposure (Figure 7A and 7B) shows that physical interactions are instrumental in determining expression of the chemoreceptor repertoire within same sex groups. However, when a solitary female is exposed to a group of males behind a cheese-cloth partition (Figure 7C) or when a solitary male is exposed to a group of females behind a cheese-cloth partition (Figure 7D), the patterns of changes in transcript abundance are distinct from those observed between isolated individuals and individuals maintained within same sex groups (Figure 6). This indicates that odor cues influence chemoreceptor gene expression between individuals of opposite sex (although a possible contribution of courtship song cannot be excluded).

### Correlated Phenotypic Plasticity of Chemoreceptor Gene Transcripts

**Figure 8:**
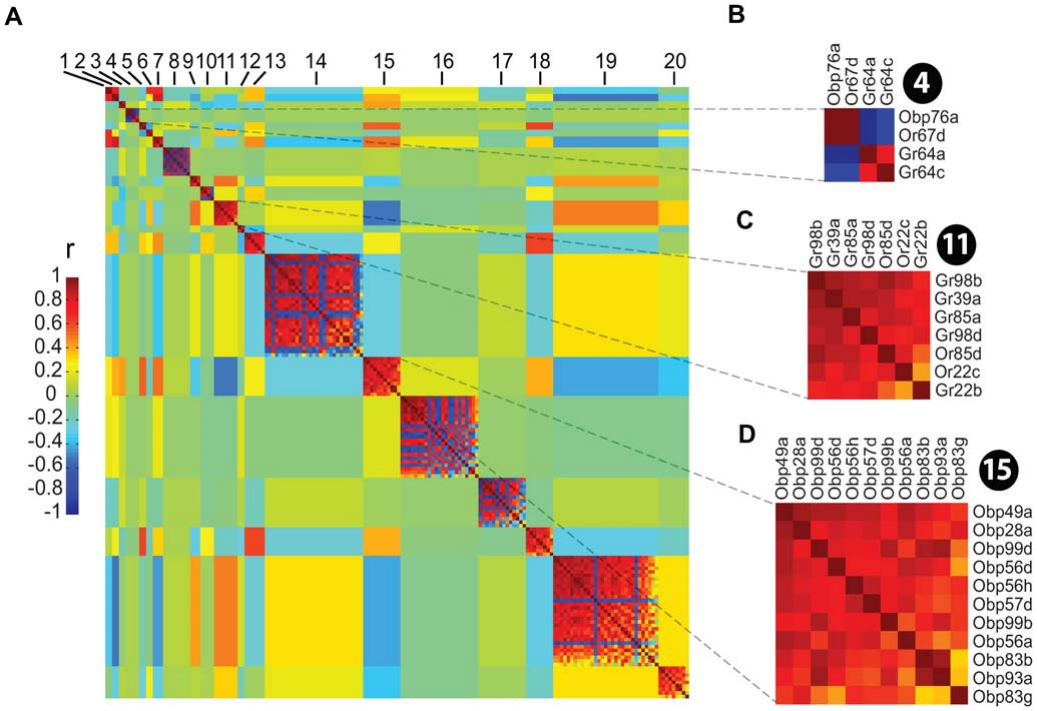
Correlated transcriptional response in phenotypic plasticity. (A) Clustering of 172 genes into 20 modules that show correlated transcriptional responses across environmental conditions. The modules populate the main diagonal and are ordered by decreasing strength from the upper left; the genes are ordered from left to right and from top to bottom as in Table S3. Color has been used to indicate strength of correlation as illustrated in the legend. Within a module, each colored square reports the correlation in transcriptional response between a pair of genes. Pairs of genes that do not share a module are given the color that corresponds to the average absolute pairwise correlation between genes from those modules. (B–D) Magnification of Module 4 (B), Module 11 (C), and Module 15 (D) with gene labels.

We noticed that environmental plasticity of expression was heterogeneous among chemosensory genes, with certain members of the chemoreceptor ensemble responding more frequently to environmental changes than others. Therefore, we decided to investigate whether groups of chemosensory genes showed correlated transcript levels across all experimental conditions. We analyzed transcript levels using the modulated modularity clustering method. This unbiased, self-organizing paradigm is based on correlations of transcript abundance levels between different conditions, and sorts transcripts into modules such that transcript abundance levels among members within each module are more closely correlated than with members outside that module [29],[30]. The resulting pairwise correlation matrix can be represented graphically such that modules of correlated transcripts are organized in a matrix, with color-coding indicating the strength of each pairwise correlation [29],[30] (Figure 8). This analysis revealed 20 covariant ensembles (Figure 8; Table S3), indicating that transcriptional regulation of the chemoreceptor repertoire is indeed modular. At the same time, however, the large number of modules and their small sizes reflect the overall heterogeneity in transcriptional regulation of chemosensory genes. Whereas genes that are members of the same cluster were by and large independently regulated (*e.g.* Figure 2), in some instances genes in close proximity to each other within a cluster appeared to co-vary in expression levels. This was the case for *Obp58b* and *Obp58c* (located 376 bp apart in different orientations; Module 3), *Obp56b* and *Obp56c* (located 855 bp apart; Module 8), *Obp83cd* and *Obp83ef* (which have a 56 bp overlap with different orientations; Module 5), *Obp 99b* and *Obp99d* (located 1298 bp apart in different orientation with one intervening gene, *Dup99B*; Module 15), *Or42a* and *Or42b* (located 4231 bp apart with one intervening gene, *Tsp42A*; Module 19) and *Or33a* and *Or33b* (located 464 bp apart; Module 14). Strong negative correlations that reflect the antagonistic regulation of chemoreceptor gene expression described above were also observed, e.g. in Module 8. *Obp76a* (a.k.a. *Lush*), which binds the courtship pheromone *cis*-vaccenyl acetate [31] shows a strong positive correlation with *Or67d*, the transcript that encodes the receptor for *cis*-vaccenyl acetate [32],[33], and a strong negative correlation with *Gr64a* and *Gr64c* (Module 4). However, based on previously published spatial expression patterns of chemoreceptor genes [6],[10],[19],[34],[35] there appears to be no overall obvious correlation between spatial expression patterns and transcriptional covariance. With some exceptions, it appears that by and large *Obp* genes are segregated in modules that are distinct from modules that contain *Or* and *Gr* genes (*e.g.* Modules 7, 8, 15), and *Or* and *Gr* genes are frequently intermixed within covariant ensembles, *e.g.* Modules 14, 19 and 20 (Figure 8; Table S3). The transcript that encodes the ubiquitous odorant co-receptor *Or83b* [6] is found in module 17. The CO_2_ co-receptor *Gr63a* [36] is in Module 16, while its counterpart *Gr21a* [36],[37] forms part of Module 13, indicating that *Gr63a* and *Gr21a* expression is not closely correlated in the range of environmental conditions investigated in this study. Interestingly, *Gr32a* and *Gr68a*, which have both been implicated in pheromone recognition during the Drosophila courtship ritual [38],[39] occur together in Module 16 (Figure 8; Table S3).

Analysis of enrichment for shared transcription factor binding motifs is restricted due to the small size of the modules. Nevertheless, we analyzed in each module 5′ untranslated regions for enrichment of 62 putative transcription factor binding motifs. We found enrichment in module 15 of a transcription factor binding site for *mirr* shared by *Obp83g* and *Obp99b* (*P* = 0.03), in module 19 enrichment of a transcription factor binding site for *pros* shared by *a5* and *Or22b* (*P* = 0.01), and in module 20 enrichment of a transcription factor binding site for *Abd-B* shared by *Or49b*, *Gr64d*, and *Gr93a* (*P* = 0.00035). However, even though some promoter regions that control cell-specific expression of odorant receptors have been identified [40],[41], transcription factors that control expression of *Or*, *Gr* and *Obp* genes remain largely unknown and may not be represented among the group of common transcription factors which we analyzed.

## Discussion

The olfactory and gustatory systems in *Drosophila melanogaster* have been well characterized [4], [6]–[12],[19], but the central problem of how ecologically relevant environmental conditions affect transcriptional variation in expression of the chemoreceptor repertoire has not been addressed previously in a systematic manner. As chemoreceptors are distributed over the entire body of the fly, including the third antennal segment, maxillary palps, proboscis, cibarial taste organs, tarsi, wing margins and the female abdominal reproductive plate, we chose to use a comprehensive analysis whole flies rather than heads. Consequently, some differences in expression between the sexes may be due to expression of chemoreceptors in non-chemosensory tissues. It is of interest to note that expression of odorant receptors in non-chemosensory organs has been observed using similar customized cDNA microarrays in both mice [42] and humans [43].

One should note that, in the absence of corresponding quantitative information about the chemosensory proteome, the relationship between transcript abundance levels and chemosensory function must be interpreted with caution. Although to date there is no evidence for posttranslational modifications of Obps, Ors and Grs might be subject to posttranslational regulatory mechanisms that may affect the amount of active gene product. Similarly, stability of mRNA has been postulated as a contributing factor to phenotypic variation in olfactory response to benzaldehyde associated with polymorphisms in the *Obp99* gene cluster in a population of wild-derived inbred lines of *Drosophila melanogaster* [44].

Here, we have shown that transcriptional profiles of chemosensory genes in *D. melanogaster* are highly plastic during early development and ageing, as a result of mating, and in social contexts. Expression of chemoreceptor genes is highly sexually dimorphic and frequently sexually antagonistic, and the extent of transcriptional responses to changing conditions is heterogeneous among the chemoreceptor repertoire. Examination of the FlyAtlas expression data base indicates that *Obp50c*, *Obp56d Obp99a* and *Gr32a* are expressed in testes, *Obp8a*, *Obp22a*, *Obp51a*, *Obp56e*, *Obp56f*, *Obp56g*, *Obp56i* and *Or59b* in the accessory gland, *Obp19c* in the ovaries and *Pino* in both ovaries, testes and accessory glands, which suggests pleiotropic functions of these chemoreceptors and may account in part for the observed sexually dimorphic expression patterns [45]. In this study we have not included an analysis of expression of the recently discovered family of ionotropic odorant receptor (*IR*) genes, which are expressed in coeloconic sensilla of the antenna and respond, among others, to water and amines [46], and which were not represented on our microarrays. It will be of interest to investigate in future studies whether these genes show similar plasticity in expression as observed for the classical chemosensory genes.

A previous study used *in situ* hybridization to detect GFP expression of odorant receptors in larvae under the control of odorant receptor-specific promoters [47] through the *GAL4*-*UAS* binary expression system [48]. This study showed expression of 25 odorant receptors in the Drosophila larval olfactory system and reported that 14 of these receptors were larval-specific [47]. Although most of the larval expressed *Or* transcripts reported in this study were also identified on our arrays, the majority of these *Or* transcripts was also detectable in adults. There was some agreement with specificity of odorant receptor expression in larvae and adults (*e.g. Or33a* was found to show larval-biased expression and 10 *Or* genes were found to be expressed in adults as well as larvae both by us and others). However, the concordance between larval specificity detected by *GAL4*-*UAS* mediated expression of GFP in olfactory tissues and direct measurements of transcript abundance on our arrays from whole flies was generally poor. This can be due to expression of chemoreceptors in adult tissues not examined by previous *in situ* hybridization or reporter gene expression, differences in detection thresholds between the techniques used, differences in the strengths of *GAL4*-linked odorant receptor promoters in larvae and adults, or possibly differences in genetic backgrounds between strains used in the two studies.

A previous study reported sexually dimorphic expression of *Obp99a* and *Obp99b* [49]. Here we showed that sexual dimorphism in expression of chemosensory genes is widespread. This is especially evident among *Obp* genes, but the apparent prevalence of sexual dimorphism among these genes may be caused by their higher expression levels compared to those of *Or* and *Gr* genes. These broad sex-dependent differences in levels of expression of chemosensory genes suggest that males and females experience, interact with, and adapt to their chemical environments differently; for example, females have to evaluate the suitability of oviposition sites.

The independent regulation of genes within clusters, which we observed, is perhaps not surprising, as it may be a necessary requirement for subfunctionalization or neofunctionalization during evolution when daughter genes of duplication events either allow refinement and/or expansion in perception of the chemical environment or the acquisition of specialized chemosensory functions. Such functional diversification is reflected in the extensive sexual dimorphism where duplication of an ancestral gene may have resulted in daughter genes with different functions in males and females [49]. Similarly, gene duplication may enable adaptations of daughter genes to specialized chemosensory needs at different developmental stages (Figure 3).

Transcript profiles change drastically after mating, not only in females but also in males. The altered transcript abundance of *Obp19d*, *Obp28a*, *Obp56a*, *Obp56g*, and *Obp99c* that we observe in mated females (Figure 5) is consistent with a previous study which compared mating-induced changes of whole genome transcript profiles on high density oligonucleotide microarrays [50]. It is of interest that some odorant binding proteins, including *Obp56e*, *Obp56f*, *Obp56g* and *Obp56i* are highly expressed in the male accessory glands [50]. Thus, in addition to a function in olfaction, these odorant binding proteins may function also (or primarily) as carriers for physiologically active ligands that are transferred from the male into the female during copulation. Chemically-induced physiological and behavioral changes in females upon mating have been well characterized [51],[52]. Biological consequences of mating in males have also been documented [23]–[25].

Both volatile chemicals and cuticular hydrocarbons signal social information in Drosophila. The gustatory receptor *Gr68a*, which is expressed in chemosensory cells in the male tarsi, has been implicated in tactile chemosensation during courtship [38], together with *Gr32a* [39]. Recognition of the courtship pheromone, 11-*cis*-vaccenyl acetate, is mediated via the odorant binding protein Lush (*Obp76a*) and the *Or67d* receptor [31]–[33]. The expression of transcripts for *Obp76a* and *Or67d* is highly correlated across the range of environments studied here, as is expression of transcripts for *Gr32a* and *Gr68a*. A large ensemble of chemoreceptor genes, however, is sensitive to the social environment and modulated based on social context and, especially, opposite sex group odor (Figure 7). The identities of the odorants that are instrumental in mediating social interactions are not known, neither are the mechanisms that give rise to alterations in chemosensory gene expression levels.

POU-domain transcription factors, such as *acj-6*, have been implicated in mediating expression of odorant receptors in Drosophila olfactory neurons [40],[53]. A phylogenetic analysis of conserved regulatory elements among sequenced genomes of 12 Drosophila species has identified regulatory elements that act combinatorially to promote or repress the expression of specific odorant receptors in the olfactory sensilla of the maxillary palp [41]. A similar array of regulatory elements acted on by various transcription factors may also regulate *Or* gene expression in the antenna. Similar elements that regulate expression of *Obp* genes or *Gr* genes have not yet been identified. It is not clear whether transcriptional regulators and their binding sites that fine-tune transcription of *Or* genes in response to environmental changes are the same as those that control *Or* gene expression during development. Our results show that such fine tuning is exquisite in that genes that are located in close proximity within clusters can undergo independent transcriptional regulation (*e.g.* Figure 3).

Elegant electrophysiological studies have provided a detailed characterization of the molecular response profiles of a large number of odorant receptors in *D. melanogaster* [18],[54]. We found that four odorant receptors with documented odorant response profiles that all respond to alcohols and aliphatic esters [54] are contained in module 14 (*Or35a*, *Or47a*, *Or85b* and *Or98a*). Together with the observation that two of the four genes in Module 4 (*Or67d* and *Obp76a [Lush]*) encode proteins that are known to respond to *cis*-vaccenyl acetate, it is reasonable to extrapolate that the observed covariance in expression may have functional significance. However, the nature of naturally occurring ecologically relevant chemical signals that are discriminated by these receptors and the functional relationships between odorant binding proteins and odorant receptors and/or gustatory receptors remain largely unknown.

Our focused analysis of the chemoreceptor gene families using cDNA microarrays that provide enhanced resolution over previously used Affymetrix GeneChips revealed that the ensemble of chemosensory genes fractionates into 20 relatively small environmentally correlated modules (Figure 8). This observation shows that plastic transcriptional responses of chemoreceptor genes to a range of environments is modular, but at the same time indicates a great capacity of groups of chemosensory genes to alter their expression levels independently under a wide range of external environmental conditions.

## Methods

### Drosophila Rearing

Isogenic *Drosophila melanogaster Canton S (B) w^−^* flies were used for all experiments and grown under standard culture conditions (cornmeal-molasses-agar-medium, 25°C, 60–75% relative humidity, 12-hr light-dark cycle) for 4–5 days, unless otherwise specified. Larvae were collected at the 3^rd^ instar stage. Sexes were reared separately after eclosion, except where indicated otherwise.

### Modulation of Gene Expression during Development

Chemoreceptor gene expression was compared between larval and adult samples, prepared by pooling an equal number of females and males. In addition, we compared young flies (10-day old) and old flies (6 week-old), transferred to fresh food every two days.

### Sexual Dimorphism and Modulation of Gene Expression after Mating

Chemoreceptor gene expression was compared between virgin females and virgin males, between virgin and mated females, and between virgin and mated males. To ensure that males had mated, we placed single males in vials with two females and collected males for microarray analysis when they were 5 days old, if females had oviposited.

### Modulation of Gene Expression by Social Context

Chemoreceptor gene expression was compared between flies reared in isolation and reared in a group of 25 same sex flies. To assess to what extent modulation of gene expression was dependent on social odor cues, we exposed single males or females to the odor from groups of flies of the same sex or opposite sex. Single flies were separated from groups of flies behind a screen of two layers of cheese cloth that prevented physical interactions (visual contact does not occur as our *Canton S (B)* strain carries a *white* mutation that renders them blind).

### cDNA Microarrays

We amplified 400–600 bp fragments from genomic DNA or cDNA corresponding to exon sequences of 50 *Obp* genes, 59 *Or* genes, 59 *Gr* genes, four genes encoding antennal specific proteins (*a5*, *a10*, *smi21F*, *Os9*), plus two housekeeping genes as positive controls (*Gapdh1* and *actin-5C*), and *Gal4* and *LacZ* as negative controls (Table S4). The identities of all amplicons were verified by sequencing and arrays were printed on a Genetix QArray2 microarray printer at the Genomic Sciences Laboratory at North Carolina State University. Experiments comparing gene expression between larvae and adult flies used arrays with four technical replicates per slide; all other experiments used arrays containing eight technical replicates per slide.

For hybridization to the arrays, fly samples were collected and frozen between 1:00 and 3:00 pm. RNA samples were extracted from 25 flies per biological replicate, subjected to one round of amplification using the MessageAmp aRNA kit from Ambion Biosystems, Inc. (Foster City, CA) and 5 µg of each RNA sample was labeled with Cy3 or Cy5 fluorescent dyes (Amersham, Pharmacia, Piscataway, NJ; cat. # PA23001 and 25001). Labeled samples were purified using the QIAquick PCR Purification Kit (Qiagen, Inc., Valencia, CA). Six biological replicates of each sample were used for each experiment and included dye swaps to control for possible dye effects. Hybridization was performed for 60 h in a water bath at 42°C in the dark.

Arrays were scanned in a GenePix 4000B scanner, and raw data gathered by GenePix Pro software.

### Microarray Data Analysis

The raw data were subjected to log2 transformation and first normalized using a mixed analysis of variance (ANOVA) model accounting for dye, array, technical replicates (nested within array), and dye×array effects, where array, rep (array) and dye×array are random effects. Residuals were then extracted from the model and used for further ANOVA analyses to assess significant differences in gene expression among the samples. We used factorial, mixed model ANOVA according to the model: Residual = μ+dye+array+rep (array)+stage/sex/condition+ε, where μ represents the overall mean value and ε the error variance, to further partition variation of transcriptional expression between dye (fixed), array (random), technical replicates nested within array (rep (array) random) and stage (or sex, or treatment) terms by gene for each experiment. We also extracted residuals from raw data across all experiments after mixed model normalization to account for technical variation for cluster analysis. We used Modulated Modularity Clustering (MMC) [29] to organize the 172 genes into modules of correlated transcripts. MMC returned 20 modules as illustrated in Figure 8. Statistically significant differences were determined following normalization of the data by mixed model ANOVA. Bar graphs in the figures show fluorescent intensities of the raw data standardized for average array intensity and dye effect by adjusting fluorescent intensities based on the overall mean fluorescent intensities across arrays and between dyes. Comparisons of chemoreceptor gene expression between virgin females, mated females, virgin males and mated males employed a loop design. The data normalization procedure and analysis were identical except for an additional *post-hoc* pairwise comparison Student's *t*-test. A detection threshold was established based on two standard deviations from the mean *lacZ* signal intensity of the negative *lacZ* control. A Bonferroni corrected significance threshold of *P*<5.68E-05 was established as a criterion for statistical significance.

## Supporting Information

Figure S1 Correlation between Cy3 and Cy5 hybridization intensities. To assess dye effects we performed hybridization with a mixture of equal aliquots from the same RNA sample, extracted from an equal number of male and female flies, labeled separately with Cy3 and Cy5. There were four replicates of each cDNA probe on the array. Note the close correlation between Cy3 and Cy5 hybridization intensities with only minor dye effects, skewed towards Cy3 at low signal intensities and towards Cy5 at high fluorescent intensities.
(1.88 MB EPS)

Figure S2 Correlation between chemoreceptor gene hybridization signal intensities on Affymetrix and cDNA microarrays. The figure shows the correlation between fluorescence intensities of an Affymetrix microarray and our customized cDNA microarray for independent RNA samples extracted from young mated adult male flies. The Affymetrix microarray data are obtained from [21]. The scatter diagram includes 174 comparisons, excluding the *lacZ* and *GAL4* genes, which were included on the cDNA microarrays as background controls.
(1.20 MB EPS)

Table S1 Genes that are differentially expressed in larvae and adult flies.
(0.09 MB PDF)

Table S2 Genes that show sexual dimorphic expression.
(0.09 MB PDF)

Table S3 MMC analysis of array data.
(0.10 MB PDF)

Table S4 Primer pairs of array probes.
(0.07 MB PDF)

## References

[1] Aloni R, Olender T, Lancet D (2006). Ancient genomic architecture for mammalian olfactory receptor clusters.. Genome Biol.

[2] Kambere MB, Lane RP (2007). Co-regulation of a large and rapidly evolving repertoire of odorant receptor genes.. BMC Neurosci.

[3] Niimura Y, Nei M (2006). Evolutionary dynamics of olfactory and other chemosensory receptor genes in vertebrates.. J Hum Genet.

[4] Robertson HM, Warr CG, Carlson JR (2003). Molecular evolution of the insect chemoreceptor gene superfamily in *Drosophila melanogaster*.. Proc Natl Acad Sci U S A.

[5] Buck L, Axel R (1991). A novel multigene family may encode odorant receptors: a molecular basis for odor recognition.. Cell.

[6] Vosshall LB, Amrein H, Morozov PS, Rzhetsky A, Axel R (1999). A spatial map of olfactory receptor expression in the Drosophila antenna.. Cell.

[7] Clyne PJ, Warr CG, Freeman MR, Lessing D, Kim J (1999). A novel family of divergent seven-transmembrane proteins: candidate odorant receptors in Drosophila.. Neuron.

[8] Gao Q, Chess A (1999). Identification of candidate Drosophila olfactory receptors from genomic DNA sequence.. Genomics.

[9] Clyne PJ, Warr CG, Carlson JR (2000). Candidate taste receptors in Drosophila.. Science.

[10] Galindo K, Smith DP (2001). A large family of divergent Drosophila odorant-binding proteins expressed in gustatory and olfactory sensilla.. Genetics.

[11] Graham LA, Davies PL (2002). The odorant-binding proteins of *Drosophila melanogaster*: annotation and characterization of a divergent gene family.. Gene.

[12] Hekmat-Scafe DS, Scafe CR, McKinney AJ, Tanouye MA (2002). Genome-wide analysis of the odorant-binding protein gene family in *Drosophila melanogaster*.. Genome Res.

[13] Nebert DW, Nelson DR, Coon MJ, Estabrook RW, Feyereisen R (1991). The P450 superfamily: update on new sequences, gene mapping, and recommended nomenclature.. DNA Cell Biol.

[14] Berenbaum MR (2002). Postgenomic chemical ecology: from genetic code to ecological interactions.. J Chem Ecol.

[15] Wen Z, Rupasinghe S, Niu G, Berenbaum MR, Schuler MA (2006). CYP6B1 and CYP6B3 of the black swallowtail (*Papilio polyxenes*): adaptive evolution through subfunctionalization.. Mol Biol Evol.

[16] Matsuo T, Sugaya S, Yasukawa J, Aigaki T, Fuyama Y (2007). Odorant-binding proteins OBP57d and OBP57e affect taste perception and host-plant preference in *Drosophila sechellia*.. PLoS Biol.

[17] Malnic B, Hirono J, Sato T, Buck LB (1999). Combinatorial receptor codes for odors.. Cell.

[18] Hallem EA, Carlson JR (2006). Coding of odors by a receptor repertoire.. Cell.

[19] Vosshall LB, Stocker RF (2007). Molecular architecture of smell and taste in Drosophila.. Annu Rev Neurosci.

[20] Anholt RR, Mackay TF (2004). Quantitative genetic analyses of complex behaviours in Drosophila.. Nat Rev Genet.

[21] Morozova TV, Anholt RR, Mackay TF (2006). Transcriptional response to alcohol exposure in *Drosophila melanogaster*.. Genome Biol.

[22] Wolfner MF (2002). The gifts that keep on giving: physiological functions and evolutionary dynamics of male seminal proteins in Drosophila.. Heredity.

[23] McKean KA, Nunney L (2008). Sexual selection and immune function in *Drosophila melanogaster*.. Evolution.

[24] DiBenedetto AJ, Harada HA, Wolfner MF (1990). Structure, cell-specific expression, and mating-induced regulation of a *Drosophila melanogaster* male accessory gland gene.. Dev Biol.

[25] Carney GE (2007). A rapid genome-wide response to *Drosophila melanogaster* social interactions.. BMC Genomics.

[26] Rollmann SM, Mackay TF, Anholt RR (2005). Pinocchio, a novel protein expressed in the antenna, contributes to olfactory behavior in *Drosophila melanogaster*.. J Neurobiol.

[27] van der Goes van Naters W, Carlson JR (2007). Receptors and neurons for fly odors in Drosophila.. Curr Biol.

[28] Ferveur JF (2005). Cuticular hydrocarbons: their evolution and roles in Drosophila pheromonal communication.. Behav Genet.

[29] Stone EA Ayroles JF (2009). Modulated modularity clustering as an exploratory tool for functional genomic inference.. PLoS Genetics.

[30] Ayroles JF, Carbone MA, Stone EA, Jordan KW, Lyman RF (2009). Systems genetics of complex traits in *Drosophila melanogaster*.. Nat Genet.

[31] Xu P, Atkinson R, Jones DN, Smith DP (2005). Drosophila OBP LUSH is required for activity of pheromone-sensitive neurons.. Neuron.

[32] Ha TS, Smith DP (2006). A pheromone receptor mediates 11-cis-vaccenyl acetate-induced responses in Drosophila.. J Neurosci.

[33] Kurtovic A, Widmer A, Dickson BJ (2007). A single class of olfactory neurons mediates behavioural responses to a Drosophila sex pheromone.. Nature.

[34] Thorne N, Bray S, Amrein H (2005). Function and expression of the Drosophila *Gr* genes in the perception of sweet, bitter and pheromone compounds.. Chem Senses.

[35] Thorne N, Amrein H (2008). Atypical expression of Drosophila gustatory receptor genes in sensory and central neurons.. J Comp Neurol.

[36] Jones WD, Cayirlioglu P, Kadow IG, Vosshall LB (2007). Two chemosensory receptors together mediate carbon dioxide detection in Drosophila.. Nature.

[37] Suh GS, Wong AM, Hergarden AC, Wang JW, Simon AF (2004). A single population of olfactory sensory neurons mediates an innate avoidance behaviour in Drosophila.. Nature.

[38] Bray S, Amrein H (2003). A putative Drosophila pheromone receptor expressed in male-specific taste neurons is required for efficient courtship.. Neuron.

[39] Miyamoto T, Amrein H (2008). Suppression of male courtship by a Drosophila pheromone receptor.. Nat Neurosci.

[40] Tichy AL, Ray A, Carlson JR (2008). A new Drosophila POU gene, pdm3, acts in odor receptor expression and axon targeting of olfactory neurons.. J Neurosci.

[41] Ray A, van der Goes van Naters W, Carlson JR (2008). A regulatory code for neuron-specific odor receptor expression.. PLoS Biol.

[42] Zhang X, Rogers M, Tian H, Zhang X, Zou DJ, Liu J, Ma M (2004). High-throughput microarray detection of olfactory receptor gene expression in the mouse.. Proc Natl Acad Sci U S A.

[43] Zhang X, De la Cruz O, Pinto JM, Nicolae D, Firestein S (2007). Characterizing the expression of the human olfactory receptor gene family using a novel DNA microarray.. Genome Biol.

[44] Wang P, Lyman RF, Shabalina SA, Mackay TFC, Anholt RRH (2007). Association of polymorphisms in odorant-binding protein genes with variation in olfactory response to benzaldehyde in Drosophila.. Genetics.

[45] Chintapalli VR, Wang J, Dow JA (2007). Using FlyAtlas to identify better *Drosophila melanogaster* models of human disease.. Nat Genet.

[46] Benton R, Vannice KS, Gomez-Diaz C, Vosshall LB (2009). Variant ionotropic glutamate receptors as chemosensory receptors in Drosophila.. Cell.

[47] Fishilevich E, Vosshall LB (2005). Genetic and functional subdivision of the Drosophila antennal lobe.. Curr Biol.

[48] Brand AH, Perrimon N (1993). Targeted gene expression as a means of altering cell fates and generating dominant phenotypes.. Development.

[49] Anholt RR, Dilda CL, Chang S, Fanara JJ, Kulkarni NH (2003). The genetic architecture of odor-guided behavior in Drosophila: epistasis and the transcriptome.. Nat Genet.

[50] McGraw LA, Gibson G, Clark AG, Wolfner MF (2004). Genes regulated by mating, sperm, or seminal proteins in mated female *Drosophila melanogaster*.. Curr Biol.

[51] Chen PS (1996). The accessory gland proteins in male Drosophila: structural, reproductive, and evolutionary aspects.. Experientia.

[52] Wolfner MF (1997). Tokens of love: functions and regulation of Drosophila male accessory gland products.. Insect Biochem Mol Biol.

[53] Certel SJ, Clyne PJ, Carlson JR, Johnson WA (2000). Regulation of central neuron synaptic targeting by the Drosophila POU protein, Acj6.. Development.

[54] Hallem EA, Ho MG, Carlson JR (2004). The molecular basis of odor coding in the Drosophila antenna.. Cell.

