# Supplementary material for: Plasticity of the Chemoreceptor Repertoire in Drosophila melanogaster
Source: PLoS Genet. 2009 Oct 9;5(10):e1000681. doi: 10.1371/journal.pgen.1000681 (PMC2750752; doi:10.1371/journal.pgen.1000681)
Supplement: Table S1 — Genes that are differentially expressed in larvae and adult flies. (0.09 MB PDF) [file pgen.1000681.s003.pdf]

Table S1. Genes that are differentially expressed in larvae and adult flies

Larva>Adult	*P-Value*	Fold Change	Adult>Larva	*P-Value*	Fold Change
Gr22d	1.0E-43	127.48	*Gr22c*	1.0E-12	2.10
Gr33a	1.0E-25	2.43	*Gr2a*	5.0E-16	2.25
Obp28a	8.0E-21	3.59	*Gr59f*	5.0E-32	5.47
Obp47a	1.0E-29	201.97	*Gr8a*	8.0E-22	4.36
Obp47b	8.0E-19	2.30	*Gr93b*	2.9E-05	2.10
Obp49a	7.0E-27	2.09	*Gr98a*	7.0E-15	3.42
Obp50d	7.0E-37	5.44	*Obp18a*	2.0E-30	6.10
Obp50e	7.0E-26	3.19	*Obp19a*	4.0E-42	39.51
Obp56a	6.0E-28	2.67	*Obp19b*	4.0E-42	29.74
Obp56b	5.0E-54	97.56	*Obp19c*	4.0E-27	13.04
Obp56c	1.0E-32	21.62	*Obp19d*	2.0E-39	12.69
Obp56h	7.0E-46	20.62	*Obp22a*	1.0E-28	55.84
Obp57d	2.0E-26	2.99	*Obp50b*	5.0E-30	4.75
Obp58b	2.0E-20	4.86	*Obp51a*	1.0E-50	85.82
Obp58c	2.0E-54	156.65	*Obp56e*	1.0E-21	2.18
Obp58d	4.0E-39	26.28	*Obp56g*	5.0E-46	83.24
Obp83cd	3.0E-30	4.61	*Obp57a*	1.0E-34	57.88
Obp83ef	1.0E-23	4.42	*Obp57b*	3.0E-28	17.51
Obp83g	1.0E-33	12.96	*Obp57c*	3.0E-42	81.58
Obp85a	5.0E-19	2.37	*Obp59a*	4.0E-31	12.58
Obp99a	6.0E-21	2.69	*Obp69a*	2.0E-31	12.42
Obp99b	2.0E-43	296.24	*Obp83b*	2.0E-18	2.13
Or30a	1.0E-16	2.38	*Obp84a*	3.0E-18	4.14
Or43a	3.0E-24	2.38	*Obp8a*	1.0E-32	12.03
Or67a	8.0E-23	2.36	*Or1a*	4.0E-25	3.95
Or83b	2.0E-21	2.12	*Or22a*	1.0E-13	2.03
Or83c	1.0E-22	2.42	*Or22b*	2.0E-14	2.13
Os9	5.3E-08	2.04	*Or45a*	2.0E-28	7.15
			Or47a	6.2E-06	2.52
			Or59b	4.0E-24	5.28
			Or63a	1.0E-25	3.58
			Or92a	7.0E-19	2.32
			Or9a	2.0E-18	2.43
