# Supplementary material for: Plasticity of the Chemoreceptor Repertoire in Drosophila melanogaster
Source: PLoS Genet. 2009 Oct 9;5(10):e1000681. doi: 10.1371/journal.pgen.1000681 (PMC2750752; doi:10.1371/journal.pgen.1000681)
Supplement: Table S2 — Genes that show sexual dimorphic expression. (0.09 MB PDF) [file pgen.1000681.s004.pdf]

**Table S2. Genes that show sexual dimorphic expression**

| Female>Male | *P-Value* | Fold Change | Male>Female | *P-Value* | Fold Change |
|---|---|---|---|---|---|
| ***Gr2a*** | 1.2E-05 | 1.93 | ***Gr22f*** | 1.0E-11 | 4.77 |
| ***Gr5a*** | 1.4E-07 | 3.40 | ***Gr59a*** | 9.0E-42 | 20.05 |
| ***Gr10b*** | 2.0E-19 | 1.67 | ***Gr59f*** | 4.0E-23 | 8.07 |
| ***Gr21a*** | 1.0E-49 | 3.88 | ***Gr64c*** | 3.0E-06 | 2.05 |
| ***Gr22a*** | 2.5E-05 | 2.03 | ***Gr64d*** | 5.0E-15 | 2.80 |
| ***Gr22c*** | 2.0E-11 | 2.33 | ***Gr64e*** | 9.4E-08 | 2.24 |
| ***Gr22d*** | 1.3E-05 | 2.48 | ***Gr65a*** | 1.0E-11 | 5.77 |
| ***Gr22e*** | 2.6E-05 | 1.28 | ***Gr98a*** | 1.9E-05 | 6.49 |
| ***Gr23a*** | 6.0E-54 | 12.85 | ***Obp8a*** | 3.0E-17 | 1.85 |
| ***Gr33a*** | 2.0E-68 | 4.14 | ***Obp19b*** | 1.8E-07 | 1.28 |
| ***Gr36b*** | 3.0E-12 | 3.25 | ***Obp19d*** | 7.3E-08 | 1.26 |
| ***Gr36c*** | 8.0E-17 | 5.39 | ***Obp22a*** | 4.0E-46 | 9.03 |
| ***Gr43a*** | 3.0E-23 | 3.45 | ***Obp50a*** | 2.0E-14 | 4.77 |
| ***Gr58a*** | 3.3E-07 | 3.36 | ***Obp50b*** | 8.0E-50 | 22.65 |
| ***Gr59e*** | 6.0E-21 | 4.09 | ***Obp50c*** | 4.0E-55 | 21.61 |
| ***Gr61a*** | 6.0E-23 | 4.14 | ***Obp50d*** | 6.0E-26 | 13.34 |
| ***Gr68a*** | 3.0E-16 | 5.96 | ***Obp51a*** | 2.0E-25 | 11.57 |
| ***Gr77a*** | 2.8E-09 | 1.36 | ***Obp56c*** | 5.1E-05 | 2.25 |
| ***Gr85a*** | 1.6E-09 | 1.59 | ***Obp56d*** | 2.0E-29 | 1.71 |
| ***Obp18a*** | 5.0E-13 | 2.32 | ***Obp56f*** | 1.0E-48 | 8.34 |
| ***Obp19a*** | 4.0E-14 | 2.48 | ***Obp56g*** | 5.0E-51 | 8.08 |
| ***Obp19c*** | 2.0E-55 | 180.71 | ***Obp56i*** | 5.0E-42 | 14.11 |
| ***Obp28a*** | 3.8E-08 | 1.69 | ***Obp57b*** | 1.0E-16 | 1.22 |
| ***Obp46a*** | 4.0E-21 | 12.53 | ***Obp57d*** | 4.0E-06 | 1.83 |
| ***Obp47a*** | 7.4E-07 | 2.31 | ***Obp58b*** | 2.0E-27 | 7.29 |
| ***Obp47b*** | 1.7E-07 | 4.10 | ***Obp58c*** | 8.0E-27 | 14.73 |
| ***Obp50e*** | 5.0E-13 | 1.63 | ***Obp59a*** | 2.0E-17 | 3.07 |
| ***Obp56a*** | 8.0E-21 | 2.42 | ***Obp76a*** | 2.0E-30 | 1.93 |
| ***Obp56e*** | 2.0E-28 | 7.21 | ***Obp83a*** | 7.2E-09 | 1.28 |
| ***Obp57a*** | 2.0E-58 | 5.34 | ***Obp83b*** | 9.0E-28 | 1.80 |
| ***Obp57c*** | 6.0E-13 | 1.47 | ***Obp93a*** | 2.0E-17 | 5.69 |
| ***Obp83cd*** | 5.0E-59 | 3.19 | ***Obp99b*** | 9.0E-48 | 9.73 |
| ***Obp83ef*** | 4.0E-40 | 2.65 | ***Obp99d*** | 2.0E-13 | 2.26 |
| ***Obp83g*** | 5.0E-05 | 1.61 | ***Or45a*** | 9.0E-17 | 4.04 |
| ***Obp84a*** | 2.0E-69 | 5.15 | ***Or45b*** | 6.0E-16 | 3.95 |
| ***Obp99a*** | 1.0E-40 | 3.01 | ***Or46a*** | 6.0E-10 | 3.64 |
| ***Or7a*** | 4.7E-07 | 2.59 | ***Or47a*** | 3.3E-05 | 2.20 |
| ***Or24a*** | 3.1E-06 | 2.13 | ***Or56a*** | 1.0E-26 | 5.45 |
| ***Or43a*** | 3.1E-05 | 2.27 | ***Or59b*** | 2.0E-25 | 10.07 |
| ***Or43b*** | 1.0E-55 | 5.33 | ***Or63a*** | 8.0E-19 | 3.59 |
| ***Or49a*** | 8.9E-08 | 1.83 | ***Or67c*** | 8.0E-10 | 4.20 |
| ***Or59c*** | 2.0E-12 | 1.72 | ***Or94a*** | 7.0E-08 | 3.36 |
| ***Or65a*** | 6.3E-08 | 1.43 | | | |
| ***Or83b*** | 5.0E-21 | 2.21 | | | |

| | | | | | | |
|---|---|---|---|---|---|---|
| ***Os9*** | 8.0E-18 | 2.08 | | | | |
| ***smi21F*** | 1.0E-13 | 1.98 | | | | |
