# Supplementary material for: Plasticity of the Chemoreceptor Repertoire in Drosophila melanogaster
Source: PLoS Genet. 2009 Oct 9;5(10):e1000681. doi: 10.1371/journal.pgen.1000681 (PMC2750752; doi:10.1371/journal.pgen.1000681)
Supplement: Table S3 — MMC analysis of array data. (0.10 MB PDF) [file pgen.1000681.s005.pdf]

Table S3. MMC analysis of array data

Gene	Module	Average Degree[1]	Degree[2]
Obp56f	1	0.97628	0.97628
Obp56i	1	0.97628	0.97628
Gr59f	2	0.95915	0.95915
Obp56g	2	0.95915	0.95915
Obp58b	3	0.91491	0.91491
Obp58c	3	0.91491	0.91491
Obp76a	4	0.89548	0.93586
Or67d	4	0.89548	0.93569
Gr64a	4	0.89548	0.89577
Gr64c	4	0.89548	0.8146
Obp83cd	5	0.89485	0.89485
Obp83ef	5	0.89485	0.89485
Gr65a	6	0.87775	0.87775
Obp50a	6	0.87775	0.87775
Obp8a	7	0.86127	0.90711
Obp59a	7	0.86127	0.84196
Obp83a	7	0.86127	0.83473
Gr22d	8	0.84434	0.8756
Obp56b	8	0.84434	0.87505
Obp57c	8	0.84434	0.87286
Obp56c	8	0.84434	0.86766
Obp19a	8	0.84434	0.86038
Obp47a	8	0.84434	0.85761
Obp57a	8	0.84434	0.79842
Obp58d	8	0.84434	0.7471
Or74a	9	0.81246	0.86926
Or82a	9	0.81246	0.81075
Or67b	9	0.81246	0.75735
Gr23a	10	0.80828	0.83235
Gr22c	10	0.80828	0.82706
Obp84a	10	0.80828	0.80657
Or23a	10	0.80828	0.76713
Gr98b	11	0.78903	0.85634
Gr39a	11	0.78903	0.84736
Gr85a	11	0.78903	0.83532
Gr98d	11	0.78903	0.80146
Or85d	11	0.78903	0.79327
Or22c	11	0.78903	0.72362
Gr22b	11	0.78903	0.66587
Or1a	12	0.77267	0.77267
Or22a	12	0.77267	0.77267
Gr10b	13	0.77114	0.82454
Gr21a	13	0.77114	0.79357
Or65b	13	0.77114	0.79189

Gr10a	13	0.77114	0.78332
Gr66a	13	0.77114	0.76062
Or67c	13	0.77114	0.67293
Gr36a	14	0.73899	0.84691
Or33b	14	0.73899	0.8377
Gr64e	14	0.73899	0.81836
Gr39b	14	0.73899	0.8135
Or94b	14	0.73899	0.81179
Or30a	14	0.73899	0.80825
Or35a	14	0.73899	0.79944
Gr58a	14	0.73899	0.79711
Or98b	14	0.73899	0.7866
Or85c	14	0.73899	0.78646
Or24a	14	0.73899	0.78493
Obp57b	14	0.73899	0.78248
Or13a	14	0.73899	0.7761
Or65c	14	0.73899	0.77282
Gr22a	14	0.73899	0.77125
Gr47b	14	0.73899	0.76015
Obp19d	14	0.73899	0.75852
Or46a	14	0.73899	0.74523
Gr93b	14	0.73899	0.74277
Or98a	14	0.73899	0.73602
Gr92a	14	0.73899	0.70441
Gr5a_Tre	14	0.73899	0.69869
Or85b	14	0.73899	0.69341
Or33a	14	0.73899	0.68639
Gr57a	14	0.73899	0.68179
Or88a	14	0.73899	0.66493
Gr64f	14	0.73899	0.63018
Obp19b	14	0.73899	0.61431
Or47a	14	0.73899	0.32024
Obp49a	15	0.7337	0.82903
Obp28a	15	0.7337	0.78494
Obp99d	15	0.7337	0.78315
Obp56d	15	0.7337	0.75847
Obp56h	15	0.7337	0.75561
Obp57d	15	0.7337	0.74906
Obp99b	15	0.7337	0.74092
Obp56a	15	0.7337	0.71767
Obp83b	15	0.7337	0.71028
Obp93a	15	0.7337	0.67891
Obp83g	15	0.7337	0.56262
Obp50b	16	0.68812	0.7942
Obp50c	16	0.68812	0.79415
Gr59a	16	0.68812	0.78853
Obp51a	16	0.68812	0.78398
Gr32a	16	0.68812	0.78212

Or45a	16	0.68812	0.7612
Obp22a	16	0.68812	0.74552
Obp50d	16	0.68812	0.73714
Obp47b	16	0.68812	0.7126
Or94a	16	0.68812	0.71181
Obp19c	16	0.68812	0.71056
Obp46a	16	0.68812	0.70693
Gr22f	16	0.68812	0.70133
Or59b	16	0.68812	0.69108
Gr68a	16	0.68812	0.68581
Gr43a	16	0.68812	0.6841
Or63a	16	0.68812	0.65644
Gr61a	16	0.68812	0.62775
Gr98a	16	0.68812	0.62122
Or71a	16	0.68812	0.62071
Or56a	16	0.68812	0.60949
Or45b	16	0.68812	0.53513
Obp57e	16	0.68812	0.36495
Obp69a	17	0.68311	0.79334
Obp85a	17	0.68311	0.78867
Gr22e	17	0.68311	0.78686
Or67a	17	0.68311	0.78044
Gr93d	17	0.68311	0.76745
Gr2a	17	0.68311	0.74634
Gr58c	17	0.68311	0.73206
Or65a	17	0.68311	0.71128
Obp18a	17	0.68311	0.69962
Or43a	17	0.68311	0.6489
Or83c	17	0.68311	0.63321
Or83b	17	0.68311	0.53475
Gr8a	17	0.68311	0.4869
Or85f	17	0.68311	0.4537
smi21F/Pino	18	0.67328	0.72713
Obp99a	18	0.67328	0.72134
Os9/Hf	18	0.67328	0.72091
Or43b	18	0.67328	0.71255
Obp56e	18	0.67328	0.70537
Obp99c	18	0.67328	0.69636
Obp50e	18	0.67328	0.61174
Gr33a	18	0.67328	0.49081
Gr59c	19	0.66409	0.7831
Or19b	19	0.66409	0.76974
Or42b	19	0.66409	0.76252
Gr59e	19	0.66409	0.75722
Gr94a	19	0.66409	0.75511
Or42a	19	0.66409	0.75475
Or19a	19	0.66409	0.74705
Or2a	19	0.66409	0.74437

Or9a	19	0.66409	0.73575
Gr77a	19	0.66409	0.73205
Gr59b	19	0.66409	0.72961
Or7a	19	0.66409	0.72145
a10	19	0.66409	0.71798
Or92a	19	0.66409	0.71301
Gr98c	19	0.66409	0.70764
Gr59d	19	0.66409	0.6871
Or22b	19	0.66409	0.68527
Gr58b	19	0.66409	0.68412
Or83a	19	0.66409	0.67207
Or59a	19	0.66409	0.6674
Gr28b	19	0.66409	0.66623
Gr97a	19	0.66409	0.65741
Or85a	19	0.66409	0.65662
Gr36b	19	0.66409	0.65297
a5	19	0.66409	0.63082
Or10a	19	0.66409	0.58843
Gr93c	19	0.66409	0.58634
Or49a	19	0.66409	0.51798
Or59c	19	0.66409	0.42858
Gr47a	19	0.66409	0.40584
Gr28a	19	0.66409	0.26819
Or33c	20	0.56638	0.64981
Or69a	20	0.56638	0.64164
Or49b	20	0.56638	0.62421
Gr63a	20	0.56638	0.62267
Gr64b	20	0.56638	0.61222
Gr93a	20	0.56638	0.6032
Gr36c	20	0.56638	0.57009
Or47b	20	0.56638	0.48975
Gr64d	20	0.56638	0.28386

[1]Average absolute correlation between all gene pairs in the module, i.e. $\frac{1}{n(n-1)}\sum_{i\neq j}\left|r_{ij}\right|$ for a module of size n

[2]Average absolute correlation between a gene and the rest of its module, i.e. $\frac{1}{n-1}\sum_{i\neq j}\left|r_{ij}\right|$ is the degree of gene i
