# Supplementary material for: Plasticity of the Chemoreceptor Repertoire in Drosophila melanogaster
Source: PLoS Genet. 2009 Oct 9;5(10):e1000681. doi: 10.1371/journal.pgen.1000681 (PMC2750752; doi:10.1371/journal.pgen.1000681)
Supplement: Table S4 — Primer pairs of array probes. (0.07 MB PDF) [file pgen.1000681.s006.pdf]

Table S4. Primer pairs of array probes

Gene	FlybaseID	5' PRIMER	3' PRIMER
Gr2a	FBgn0027796	GGACACGCTGAGAGCTCTGG	CGCACGAGCTGAGTGGCG
Gr5a/Tre	FBgn0003747	GTGCAATCGTGCTGTTCG	GATGCTCAGCAGGTGTTC
Gr8a	FBgn0030108	CGTGTGGCTGACGTATCT	CGTATCGGCTGATGCAGG
Gr10a	FBgn0045502	GAGCCACCGCCTCCAGCTC	CTTCAAGTTCTGCCTGATC
Gr10b	FBgn0030297	GGTCGCTGTAGGTGTCGTGC	CTACAATCCCACTCGGCGG
Gr21a	FBgn0041250	GGTAGTGCCGATGCTGAA	CAGGAGAAGACGCACAGCG
Gr22a	FBgn0045501	CGGTCAGGATCTACGCTA	GATCCTGGGTCCTAGGAC
Gr22b	FBgn0045500	GGACACCGACATGGTAGTGC	GGATCCTGGCTACTCGGT
Gr22c	FBgn0045499	GTTGCCAGGCAGTCCGTAG	CATCACGTAGCGACTTGCAG
Gr22d	FBgn0045498	GGCAGTCGTAGCAATCGG	CCAGTCTCCTTATTCTGATG
Gr22e	FBgn0045497	GGATGCTATCGACCTGTTC	GTCCATCCGCTCAGACTC
Gr22f	FBgn0041249	CTTGAGGAGCTTAGATGG	GACCCTCTACGCATCCTG
Gr23a	FBgn0041248	GGTGCAGCACGTCATGGA	CGCAGTGAGCAGTCCAAT
Gr28a	FBgn0041247	GAGGTAGCACGTGGCAGC	CGCTACTCACATCGCCTAC
Gr28b	FBgn0045495	CGAATGCTGCTGCAGAGG	GGACGTGCAACTCCAGAC
Gr32a	FBgn0041246	CGTGCAAGGCGAGATCAC	CTCCTTCGACTTGGCATAC
Gr33a	FBgn0032416	CACGTGTCGGTCATCTCGC	GCAGCATAGTCGAAGCAC
Gr36a	FBgn0045487	GCTCAAGACAGAGGTGCG	GCAGCAGATGAACTGCGA
Gr36b	FBgn0045486	GTGGATGCATTGGACCG	CTCCAGTCGAATGTCCAAG
Gr36c	FBgn0045485	GGCTTGCAGTACTGGATG	GGCTTCTTCGAGGCGGAC
Gr39a	FBgn0041244	GCAGATCAGCCGTGTCCTG	CTAAGTAGGCTTGCAAGG
Gr39b	FBgn0041245	CCATCAGATGACACTCTAG	CCTTTCATCCGTACCTC
Gr43a	FBgn0041243	CGGAGAAGTCAGCATCTAC	CAATGCAACCAGTGCCATC
Gr47a	FBgn0041242	CAGCTTGCAGTTCTCAGA	CTGCGATGGTAGCAGTCT
Gr47b	FBgn0041241	GAGTGCCTCAGTTGCTCGGTC	GGCAGATAACGTACACG
Gr57a	FBgn0041240	CGAACTGGATGAGTATGAGC	GCTGCAGCAACTGCTACAGC
Gr58a	FBgn0041239	CTACTGACCGTCCTGCCCT	CTTCTCGGACTAATCGCTG
Gr58b	FBgn0041238	GATGCTGCTATTGCTGTAC	CCTTAGAGCCATCGCTGT
Gr58c	FBgn0041237	CGTGGTGGCTCTGACCAA	CACTGGAAGATGTCAACG
Gr59a	FBgn0045483	GCAGTCGCGGATTACTCG	CTCCAAGTCCATCGATTG
Gr59b	FBgn0045482	CGTGACGCTCATCATGTTGC	GCTGTCTTGGTGAGGCAG
Gr59c	FBgn0041235	GAGTGATCACTGAGCTTAG	GGTGCAGGTGATTGGGCT
Gr59d	FBgn0041236	GATCACGAGCAGGTTATTCC	GGAGCTGGTCAGTCGATG
Gr59e	FBgn0041233	CTGCAGACCTCCAACTCG	GACGTGGTCAACTATGAC
Gr59f	FBgn0041234	GAGTTGGATGATTCTGTGG	GAGCAGTAGGATGGAGTA
Gr61a	FBgn0035167	GGTGGACATTCTGATGCT	GATCAGCGAGTACCAGAAG
Gr63a	FBgn0035468	GGTCAACTTGCTCAGTCG	CACATTGTGCGCTCCCTG
Gr64a	FBgn0045479	CGAATCTGGACGAGAACGG	CTCCAGTAGCTCGCACATC
Gr64b	FBgn0045478	CTGGCATGGAGTGGAGCA	CAGCGAATCCTAAGGAGT
Gr64c	FBgn0045477	GCTCTGATCCTGTTCGTC	CGAATCCTCTGCCAGAAT
Gr64d	FBgn0035486	GCTGAGGAGCCATTTGTC	GGTATACAGCTGAAGGCC
Gr64e	FBgn0045476	CATGATCACCATCGTGGC	GTGGTCGCTTGGACTCATC
Gr64f	FBgn0052255	GCTGGTCTTGATAGTGGC	CTCAGCAACTGGACGCAA
Gr65a	FBgn0041232	GGATCTATTGAGGACACT	GTAGTGCTCATTGCCACG
Gr66a	FBgn0035870	CTCAGGTAGATGAGGTAC	GAGGAACTGGTGGACACG
Gr68a	FBgn0041231	GAGCAACCTCATAGAGGT	GATAGGTAGTAACAGCCG
Gr77a	FBgn0045474	GATCATCCTTGAGTATCTG	CTCAGCTCGGATACATTC
Gr85a	FBgn0045473	CAGCGACAGCCATTCGAT	CCATGAACTACCTGCTGT
Gr92a	FBgn0045471	CGTGTGGCTGACGTATCT	GCCAATCAAGGCAATTAGT
Gr93a	FBgn0041229	GTGGCGCTGCATTGTGGT	CACACTCGTCCAACTCAT
Gr93b	FBgn0045470	GCAGAATGGCGTGGTAGGAA	GCTGTCTGATCTGCAGCG
Gr93c	FBgn0045469	CATTCGTGAACTACTGACC	CTGGTCTACGAACTGTAC
Gr93d	FBgn0045468	GCGTCTGCGATACACGAT	GGAGTTCTCTATGCTGAG
Gr94a	FBgn0041225	CGCCACCACTCTGTACAG	CCACCGTTATTCCGAGCA
Gr97a	FBgn0041224	CCTCGTTGATGTAGTGAT	CAACTATGTGACCCAATG
Gr98a	FBgn0039520	GCACCATCGACCTGAGCA	GTGCTCAACTCTGCTGAG
Gr98b	FBgn0046887	GATGGTACTTGATGACTG	GAGCTTGAAGCATGTCCT
Gr98c	FBgn0046886	CGACTCCGACTGACGCTT	CAGCTGTAGGCAAGCATC
Gr98d	FBgn0046885	GCCTGTGGATAGTGTTGC	GAATGCAGAACTCGCTGT
Obp8a	FBgn0030103	GGAGATCACAGATCGGTT	GCTATGTCACAATAGCTAG
Obp18a	FBgn0030985	CTGTGATGAAGCCCATTG	GCATAGCTGTACTATGGA
Obp19a	FBgn0031109	GGTCTGGAGTCTGGAATC	CACCTGTTCCGAATGCCA
Obp19b	FBgn0031110	GATGCAGTGCAGCCGAATG	GGAGAACATGCAATGGTG
Obp19c	FBgn0031111	GCTGGCGACTGATGCACTGG	GAAGCCATCCACTCCAGT

Obp19d/Pbprp2	FBgn0011280	CCAGAGCTGTTGATCGAT	CCTGTTGATCTCCTCGTG
Obp22a	FBgn0043539	GTGGCACATGTCGCGTTG	GCGAGTGTTGCTGGCTTT
Obp28a/Pbprp5	FBgn0011283	CCTCGCAGTGATCATCCG	CACCGACCTAGCATCATG
Obp46a	FBgn0033508	GCGCTTGGTGGGACAGTG	GTGCTCCCAACTGTTCGC
Obp47a	FBgn0033573	CGAGTTCTAGTGCTATTGC	GCCACGAATCGCGTGGCA
Obp47b	FBgn0033614	GCTCAAGATCACGATAACG	GCCAAGCCACGATTGATTG
Obp49a	FBgn0050052	CATGTCAGTGCACTGCTG	CGCAGCTATTACTGCTCG
Obp50a	FBgn0050067	CAAGCGAGCATACGCACA	GGCGTATACTTGTTGCTT
Obp50b	FBgn0050073	GTCTTCGGTGCTGCATCT	GTGCAACTTGACAGCAAT
Obp50c	FBgn0050072	CCTGCTGATCTGCTCATT	GCGATGACTAGAACCACG
Obp50d	FBgn0050074	GCTTCACAAGCTGACTTG	GCGAATGCTGTATTCCCT
Obp50e	FBgn0033931	GCAACGGAACATGAGGCA	GGAATGTTCGTTGGCGTC
Obp51a	FBgn0043530	CACCAAGTAACCGAGCTC	GCTGTCACTACGCTGTCA
Obp56a	FBgn0034468	CTCAACATGAACTCCTAC	CCCGAATCACAATTTGCC
Obp56b	FBgn0046880	GTCACAGGTGGCGGCTGA	CTTATCTACTTGTTGGTTG
Obp56c	FBgn0046879	GACTTAGCGGTGGTCATT	GTTCCAGATCCGCCAAATG
Obp56d	FBgn0034470	CGGAGATTTGGCGCTTAG	GCAGGAGGGAATCACCAA
Obp56e	FBgn0034471	CAGCAGATTACGATCAGC	GCACGCCTATTTGGGAAG
Obp56f	FBgn0043533	TGCTATCTGGCTCCAAGC	GCTTGGTAATGGACTGAT
Obp56g	FBgn0034474	GGCAGTGTCACAGGCATT	GGCTACATTCGCATTGAC
Obp56h	FBgn0034475	CCGGACTTTCGCCAAATA	CTGGAATGGCCTTGTGCT
Obp56i	FBgn0043532	CACCTGCTGTGCATTATTG	CCTCTGTGATTTCGAGTT
Obp57a	FBgn0043535	GGAAAGCCAACCCTTTGA	AAACAGATCCGCCTGCTG
Obp57b	FBgn0043534	GTTCATCTACAGACTTGT	GCACACATCACCAGTCTA
Obp57c	FBgn0034509	GAGGTGCTACTGTGCTAG	GACCTCATCGCTCTGCTC
Obp57d	FBgn0043536	TCTTGCCTTAAACAATTGAA	ATTGCCGATTCTAACGATCC
Obp57e	FBgn0050145	CGCAAGCTTCACATCCTTG	CCAACTTACACTGTGTTTG
Obp58b	FBgn0034768	GGACTCGTGGCTGTCCGA	CAGCACATCCTTGCACTT
Obp58c	FBgn0034769	GGCACTTCTTGCTGAAGG	GATTGCGAGAATACGGAG
Obp58d	FBgn0034770	GGACGTTCCTAATTCTGG	CGCACATATCCTTAGTTCC
Obp59a/Obp58a	FBgn0034766	GCAACTGTATGCATCGCC	GAAGAGCATGTTGCCGTG
Obp69a/Pbprp1	FBgn0011279	GCTGCGAATGCGATGCTT	GCTCTTGGGAATCGTGGA
Obp76a/lush	FBgn0020277	CATCATTTCCGGGGGAAC	GGCGTGCGAGTGTGTTAC
Obp83a/Pbprp3/Os-F	FBgn0011281	CATGCACAACTCGCCTAC	CCTTGTCGCTGCTCAGCG
Obp83b/Os-E	FBgn0010403	GTAGTGGACAGGATCAGC	CAAATACCCACTGATACTAC
Obp83cd	FBgn0046878	CGAACGTCTAGAACGATTC	GCGTAGCAGGTGAACTGT
Obp83ef	FBgn0046876	CTACTGCTCTTCGTTGTCC	CGGTATTCATCTTGGACG
Obp83g	FBgn0046875	CCTGTAAGGCGTACAGTG	CACATTCGCACTTGAGCC
Obp84a/Pbprp4	FBgn0011282	GATAAGTCCGAGATGTGAC	GAGCTTGTGCTGTCATTG
Obp85a	FBgn0037589	GCTCGCATTTCCGATACG	CCCTGGGTCGGTGGTATT
Obp93a	FBgn0038859	CCACATCGCTGTGCAATC	CTGCGATCACGCAAAGCA
Obp99a	FBgn0039678	GTCTTCGCTCGATCGCTG	GAATCGAGAAGGAATGCT
Obp99b	FBgn0039685	GACAAGCGATCAGATGGT	CATACATCAGCATGAAGG
Obp99c	FBgn0039682	CATCATGCACTGGTGTCC	CTCAGTTCAGAACCTCAAC
Obp99d	FBgn0039684	GAATCACTTGAGACTGGAG	GACTCATGAAGTCCTTGAC
Or1a	FBgn0029521	GATGCACGGACTACAGAT	CCACAAATGCGATCTCCA
Or2a	FBgn0023523	CAGTGCTGTGTACTACCAC	CGTGAGCAGGCAGAGAAAC
Or7a	FBgn0030016	CACTGGCACTGGAAGATG	GCTATGCCATCTACTCCT
Or9a	FBgn0030204	CATCATGATGTACGACACG	GCACTCTGCGTGGACTCG
Or10a	FBgn0030298	GCTGAACGAGTCGACGTT	CACCCTTCAATATGACTA
Or13a	FBgn0030715	CGTGGTACGTCATTGCAT	CTGCGATTCGTTGGTGTA
Or19a	FBgn0041626	CAGCAGAGTAAGCATGGT	CACTTGTCCTCAATCTGA
Or19b	FBgn0062565	CCTTCCACATCTGCATCT	CTGGTGGTCGTGGATGTA
Or22a	FBgn0026398	CTCGAGATTGGAGTCAAC	GACCCAGTACAGTACCGA
Or22b	FBgn0026397	GAGACAGGAGGCTAAGATG	CTGCATAGATACGCAGGAC
Or22c	FBgn0026396	CCATCTGCTCTTCGTGTT	CCTGCGAATGTCCTGCTG
Or23a	FBgn0026395	GGTACTGGAGTTGGTCGA	GCAGTTGACCAGATCCTG
Or24a	FBgn0026394	GCACAAGTGTAGACCACA	CACTGGCAACGCAACTAAC
Or30a	FBgn0032096	GGTAGCACTCTGAAGCTA	GCAACACTCGACACATGA
Or33a	FBgn0026392	CAAGTCTTCAGGAGTCTG	GTTGGCTAAGATGTAAAG
Or33b	FBgn0026391	GGATGTCTGCAAGGGTCT	GTGATCCTCGATGCTTTCG
Or33c	FBgn0026390	GGATCTGCATGCGATTGC	GACCACAAATGGACATGTC
Or35a	FBgn0028946	GTCGCTGCATCGAAGTGA	CAAGAGGGACTTGCAGTTG
Or42a	FBgn0033041	GTAGGAAGTACATGAGTG	GCGATCTTCATTGGAAGG
Or42b	FBgn0033043	CTTATGTTGCTGCTCATGG	CTGGCAGCTGTACAATCC
Or43a	FBgn0026389	GAGTTCCTGCCTCGTACC	GTCGGTGCTCTGGTATCG
Or43b	FBgn0026393	CTGCAGGAACTGGATGAC	CTGGACGGACTACTGCAC
Or45a	FBgn0033404	CTCATCCTGTCCTTGATC	CTCCGCCATTCAGATCCT
Or45b	FBgn0033422	CGTGGCTTCACCTCTTGT	GGCGTACACAGTGACCTG
Or46a	FBgn0026388	CTACTGCTATCAGTGTCTG	CAAGGTCGAGTGAAGGCG

Or47a	FBgn0026386	CTGCTGTCAGCTTGGATAC	CATTGTGATGTAGGACATCG
Or47b	FBgn0026385	CTGGTGGAAGCGAACCAC	TACATGCATTTGCGTTGC
Or49a	FBgn0033727	GCTATCTGCTTGTGCGAG	GCTTGAGACGCACATCAT
Or49b	FBgn0028963	GAGTACGAGAGTCCGTAC	CCCTCATCATCATGAACA
Or56a	FBgn0034473	CCATCTCCTCCACTCGCT	GCATCGATTTGGCTTAGCTG
Or59a	FBgn0026384	GAAGTACATCCGTGCCAT	GGATGTGCTGGAGATGGA
Or59b	FBgn0034865	CGATCAGCATGTTGCAGG	CGACAGCGATCGCGAGAGG
Or59c	FBgn0034866	CGATGATCCTGAATGCAG	GCTCGGACTGAGCCTCAC
Or63a	FBgn0035382	GCGTGAGTAGCTTGGCAT	GTGACCTCATAAGTCCCA
Or65a	FBgn0041625	GTCAATCTGGGACGAGATC	GGTTGCAGCGATCTGTAAG
Or65b	FBgn0041624	GTGATCAGCGATCTGGAC	GTCCTTCCGAGCCTCCAC
Or65c	FBgn0041623	CCTATCGTTCCTCTTGGC	GCACACAAGCCAAGTCAG
Or67a	FBgn0036009	TGGATAACGTCGCGGAAA	CAACCAGGAGTCGCGAAA
Or67b	FBgn0036019	GTAGAGCAGCATGCAGATC	GGAACTGCTGAGCTCAGT
Or67c	FBgn0036078	GATACGACATGCTGATGAC	CTGGATCATGTACTGGGA
Or67d	FBgn0036080	GAAGCGAATCCGCATTACAT	AGCCGCAAAAGGTGTAGAGA
Or69a	FBgn0041622	CGTAGCACGCATCATCAG	CTCGAACTCACAGCAGTTG
Or71a	FBgn0036474	CTGTTGCTTGAGTCGCTG	CAGCATACCGCCGATGTC
Or74a	FBgn0036709	CTCGAGTTCAGTTGGATG	GGTGCTCAACTTCTGGGT
Or82a	FBgn0041621	CCGATAGTACCGCACTCA	GCAACAACGACGACAGTG
Or83a	FBgn0037322	CACTGCGAACATCCTTCA	GTGGCCTGCATATGGTGC
Or83b	FBgn0037324	CACGATCTGGACGAAGGT	CTCGCTGGACACCTACAG
Or83c	FBgn0037399	CCACTCAACTCATACCAG	CTACGTAGTCACGTACATG
Or85a	FBgn0037576	GCAGCCTTATGCAATCGTG	CGCAATCGCTGCTCAGCT
Or85b	FBgn0037590	CGAAGCAGATGACGAACG	GGAAGCAGTCACTGCGTT
Or85c	FBgn0037591	GACGATAGCGAATGTCAG	GTGCTGCTGTTCTGTCAA
Or85d	FBgn0037594	CCTACGATCACAAGTACAG	CATGTCAGCTGCTAGCTG
Or85f	FBgn0037685	GTGCAGTACAGCTACGAG	GATGCTGATGTAGATCCA
Or88a	FBgn0038203	CAGCATGGTGAACGCATC	GTCCCTGCATCACATTGA
Or92a	FBgn0038798	GCAGTTCGGTCTCACAGT	CACGAGCGATGCGGCAAT
Or94a	FBgn0039033	CTATCGCTTCCTGCTCCA	GAAGATGGCACGCAACTGC
Or94b	FBgn0039034	GTACTGCACCTTCCACTG	GGTCTGGCTTTCTCTTCG
Or98a	FBgn0039551	CGAGACAGCACTTATGCT	GTCAATCTGTCAGCTATG
Or98b	FBgn0039582	GCAGCATCTGCTGCATTC	GGCAATCTGGAATAGGGC
Os9/Hf	FBgn0014000	CATGTTGATCAGACAGTC	GTACTTCGAGGAGCTCGA
smi21F/Pino	FBgn0016926	GAGCACTATGCACATTAG	GTTGTGCAGCGTTGAGTC
a5	FBgn0011294	GGATAACGACGAGAATGTC	CACTACTCACTGACACCA
a10	FBgn0011293	GAAGCTTCTCTGCAGTTT	GCATACGATCGATTCGAA
Act5C	FBgn0000042	GTTGCTGCTCTGGTTGTC	GGTCAGGATCTTCATCAG
Gapdh1	FBgn0001091	CTCCTCGACCTTAGCCTT	CAGAAGATCACCGTGTTC
Gal4	/	TGACGAGCAAGGTCTTCG	GCCAAGGGTGTTCCTTCA
LacZ	/	CGATCGCGTCACACTACG	CCGTGGCCTGATTCATTC
